# Application of long single-stranded DNA donors in genome editing: generation and validation of mouse mutants

**DOI:** 10.1186/s12915-018-0530-7

**Published:** 2018-06-21

**Authors:** Gemma F. Codner, Joffrey Mianné, Adam Caulder, Jorik Loeffler, Rachel Fell, Ruairidh King, Alasdair J. Allan, Matthew Mackenzie, Fran J. Pike, Christopher V. McCabe, Skevoulla Christou, Sam Joynson, Marie Hutchison, Michelle E. Stewart, Saumya Kumar, Michelle M. Simon, Loranne Agius, Quentin M. Anstee, Kirill E. Volynski, Dimitri M. Kullmann, Sara Wells, Lydia Teboul

**Affiliations:** 1 The Mary Lyon Centre, MRC Harwell Institute, Didcot, Oxon OX11 0RD UK; 2 Mammalian Genetics Unit, MRC Harwell Institute, Didcot, Oxon OX11 0RD UK; 3 Institute of Cellular Medicine and Ageing and Health, Newcastle University, Framlington Place, Newcastle upon Tyne, NE2 4HH UK; 4 UCL Institute of Neurology, University College London, London, WC1N 3BG UK

**Keywords:** Allele validation, Conditional, CRISPR/Cas9, Homologous recombination, Mouse, Mutant, Long single-stranded DNA

## Abstract

**Background:**

Recent advances in clustered regularly interspaced short palindromic repeats (CRISPR)/CRISPR-associated protein 9 (Cas9) genome editing have led to the use of long single-stranded DNA (lssDNA) molecules for generating conditional mutations. However, there is still limited available data on the efficiency and reliability of this method.

**Results:**

We generated conditional mouse alleles using lssDNA donor templates and performed extensive characterization of the resulting mutations. We observed that the use of lssDNA molecules as donors efficiently yielded founders bearing the conditional allele, with seven out of nine projects giving rise to modified alleles. However, rearranged alleles including nucleotide changes, indels, local rearrangements and additional integrations were also frequently generated by this method. Specifically, we found that alleles containing unexpected point mutations were found in three of the nine projects analyzed. Alleles originating from illegitimate repairs or partial integration of the donor were detected in eight projects. Furthermore, additional integrations of donor molecules were identified in four out of the seven projects analyzed by copy counting. This highlighted the requirement for a thorough allele validation by polymerase chain reaction, sequencing and copy counting of the mice generated through this method. We also demonstrated the feasibility of using lssDNA donors to generate thus far problematic point mutations distant from active CRISPR cutting sites by targeting two distinct genes (*Gckr* and *Rims1*). We propose a strategy to perform extensive quality control and validation of both types of mouse models generated using lssDNA donors.

**Conclusion:**

lssDNA donors reproducibly generate conditional alleles and can be used to introduce point mutations away from CRISPR/Cas9 cutting sites in mice. However, our work demonstrates that thorough quality control of new models is essential prior to reliably experimenting with mice generated by this method. These advances in genome editing techniques shift the challenge of mutagenesis from generation to the validation of new mutant models.

**Electronic supplementary material:**

The online version of this article (10.1186/s12915-018-0530-7) contains supplementary material, which is available to authorized users.

## Background

Classical gene targeting employing embryonic stem cells has long been the principal method to introduce complex alleles into the mouse genome [1]. More recently, microinjection of an RNA-guided engineered nuclease (RGEN) together with a single-stranded oligodeoxynucleotide (ssODN) has revolutionized our ability to direct mutations in vivo [2]. However, clustered regularly interspaced short palindromic repeat (CRISPR)/CRISPR-associated protein 9 (Cas9)-aided knock-ins of larger cassettes or loxP sites directly into one-cell mouse embryos [3, 4] were breakthroughs that have remained technically very challenging [5]. Equally, CRISPR/Cas9 reagents and ssODNs have become widely used for the introduction of point mutations in one-cell embryos (see examples in [6–8]). However, particular locations within genomes, including sequences that are highly conserved and/or repeated, regions with a low number or absence of -NGG tri-nucleotides or sequences without active single guide RNA (sgRNA) close to the target can represent a barrier to the generation of specific mutants [9].

Miura and colleagues [10] first proposed long single-stranded DNA (lssDNA) molecules, larger than standard chemically synthesized oligonucleotides, as an efficient alternative donor template for RGEN-aided homologous recombination (HR). The authors recently extended the method to the creation of conditional alleles and tag insertions, showing the generation of sequence-perfect alleles [11]. We and others documented that CRISPR/Cas9-aided genome editing can give rise to unexpected allele rearrangements (“illegitimate repairs” [7], “KI + indels” [9, 12]); therefore, thorough validation of new models is essential to ensure reproducibility of the studies employing these models [12–15]. However, limited data are available on unexpected events arising from the use of lssDNA and the associated requirements for the quality control (QC) of new models. With our extensive experience in the generation of conditional alleles through large-scale mouse model production [16, 17], we have developed a strategy for validation of these alleles.

Here, we have extended the application of lssDNA to the generation of more conditional knock-out (cKO) alleles directly in the embryo. We also produced point mutations where the desired nucleotide change is remote from active CRISPR cutting sites, which so far had proved technically challenging with the available protocols. Although not all attempts were successful, we confirm that new designs employing lssDNA indeed facilitated mutant production for cKOs and particular point mutations that had previously been challenging to generate. Furthermore, we show that novel point mutations and imperfect and/or off-target donor integration(s) can occur in the process of mutagenesis. This work emphasizes the importance of a comprehensive strategy for the QC of new mutants. We conclude that the utilization of lssDNA donor templates shifts the challenge of mutagenesis from generation to the validation of new mutant models.

## Results

### Generation of a conditional knock-out allele

#### Production of F_0_ animals

Proof of principle for the RGEN-aided generation of conditional alleles employing two CRISPR/Cas9 cuts and two separate ssODN templates as donors was published in the early days of CRISPR/Cas9-aided mutagenesis [3]. However, the use of this strategy for allele generation has not flourished in the literature in the same way as other CRISPR-directed mutagenesis applications [18]. This is most likely because its success requires two concurrent events of homology-directed recombination occurring on the same allele, which remain less frequent than non-homologous end joining (NHEJ) events [5]; this is in keeping with our own experience of the approach (see examples below). We therefore decided to pilot the use of lssDNAs as a possible alternative to ssODN donors.

**Fig. 1:**
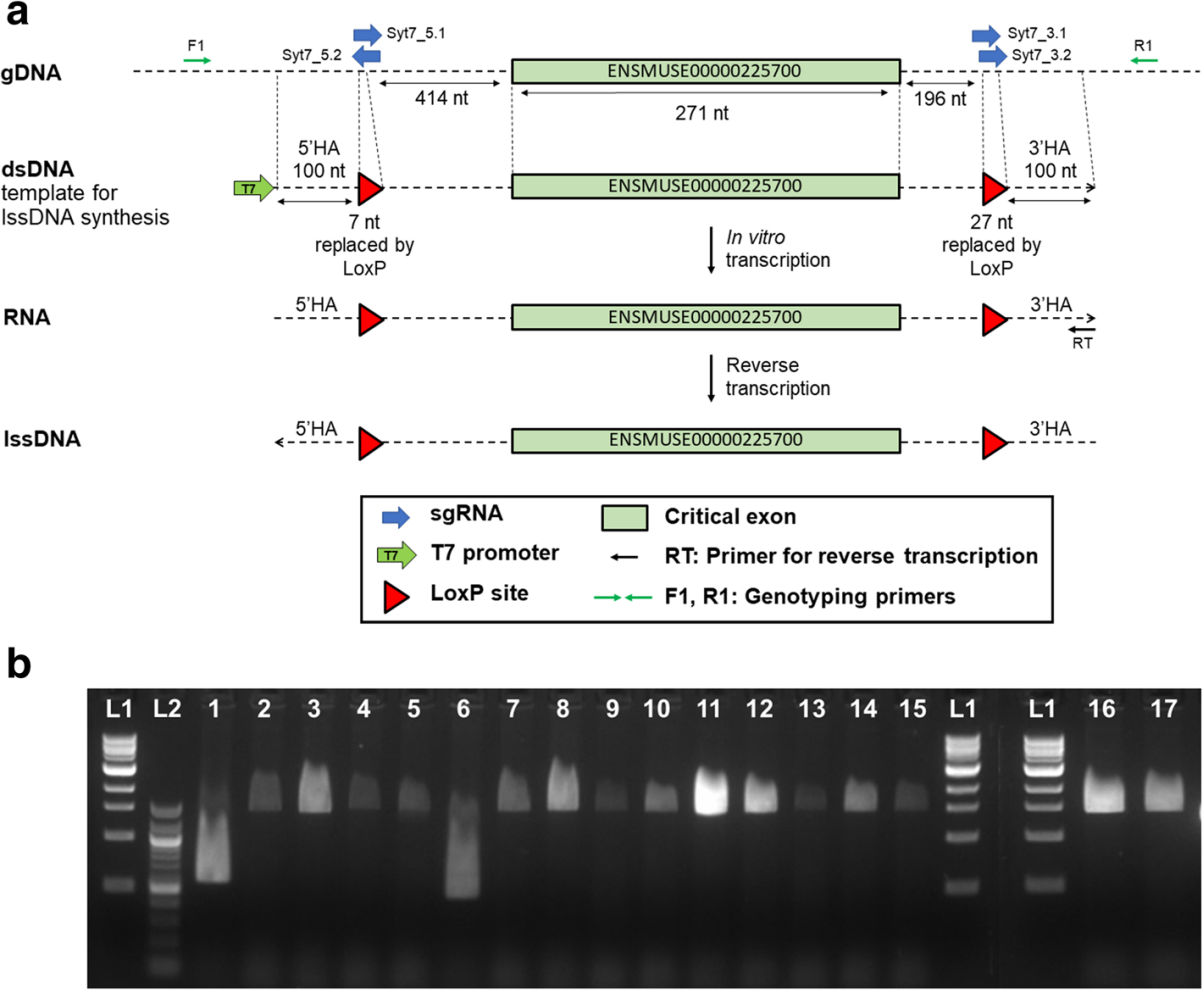
Generation of a *Syt7* floxed allele. **a** Diagrammatic representation of the genomic sequence with the Syt7 critical exon highlighted, the corresponding template for lssDNA synthesis and the position of sgRNAs for in vivo delivery together with the primer locations used for reverse transcription and for genotyping. Note loxP sites in the lssDNA prevent reprocessing of repaired alleles by CRISPR-Cas9 complex. Diagram shows the process for the generation of lssDNA through in vitro transcription and reverse transcription. *HA* homology arm. **b** PCR products amplified from genomic DNA extracted from the 17 F_0_ born from the microinjection session using Syt7-F1 and Syt7-R1 primers. L1 = 1 kb DNA molecular weight ladder (thick band is 3 kb). L2 = 100 bp DNA molecular weight ladder (thick bands are 1000 and 500 bp). Sequence trace data derived from animals Syt7-4 and Syt7-8 are displayed in Additional file 2: Figure S1.

As a first test case, we aimed to generate a conditional allele in *Syt7* by flanking the critical exon ENSMUSE00000225700 with loxP sites (Fig. 1a). This exon was chosen as defined by Skarnes and colleagues [19]. Specifically, the exon is common to the majority of coding transcripts in the gene, and its ablation results in frame-shift transcripts. Two pairs of sgRNAs were designed, centred on each of the genomic sequences to be interrupted by loxP (Fig. 1a), and synthesized to enhance the likelihood of simultaneous cuts on both sides of the same allele. A lssDNA donor corresponding to the floxed allele was generated as per Miura and colleagues ([10], and see Methods). Specifically, a double-stranded DNA template including a T7 transcription promoter followed by the 1149 bp sequence of the donor was obtained commercially (gBlock®, Integrated DNA Technologies (IDT); Fig. 1). A lssDNA was synthesized by in vitro transcription (IVT) and reverse transcription (detailed in Methods). The sgRNAs and lssDNA (the sequences are provided in Additional file 1: Table S1) were co-injected with Cas9 mRNA into one-cell embryos. One hundred thirty-eight injected embryos were re-implanted in pseudopregnant females. Seventeen pups were weaned and ear biopsies taken for screening of new alleles (the numbers are summarized in Additional file 1: Table S2, *Syt7*).

**Table 1:** Generation of conditional knock-out mice using lssDNA

		Number of F_0_s (with)							Number of lines with:				
Gene name	MS^a^	Biopsied	Mutation	cKO alleles	Illegitimate repair	Exon deletion	Indels	Progeny^b^	GLT cKO	GLT exon deletion	GLT illegitimate repair	Additional insertion(s) detected/tested by ddPCR	QC pass
*Syt7*	1	17	10	2	1	3^c^	5	2 (+ 2)	2	0 (+ 2)	1 (+ 0)	1 (+ 1) / 2 (+ 2)	2 (+ 2)^f^
*Ikzf2*	1	17	5	1	1	3	5	1 (+ 1)	1	1 (+ 0)	1 (+ 1)	0 (+ 0) / 1 (+ 1)	1 (+ 1)
	2	6	4	0	1	3	4	0 (+ 2)	–	– (+ 2)	– (+ 0)	– (+ 2) / – (+ 2)	– (+ 1)^f^
*Syt4*	1	26	16	0	10	1	11	0 (+ 1)	–	– (+ 1)	– (+ 1)	– (+ 0) / – (+ 1)	– (+ 1)
	2	40	16	1	9	1	9	2 (+ 1)	1	0 (+ 0)	2 (+ 1)	0 (+ 0) / 1 (+ 0)	1 (+ 0)
*Usp45*	1	19	7	1	0	2	6	1 (+ 1)	0	0 (+ 0)	1 (+ 0)	–	0 (+ 0)
	2	6	1	0	1	0	1	0	–	–	–	–	–
*Rapgef5*	1	30	13	1^d^	5	6	8	0 (+ 2)	–^d^	– (+ 1)	– (+ 0)	0 (+ 0) / 0 (+ 1)	– (+ 1)
*Cx3cl1*	1	3	3	0	3	1	3	0	–	–	–	–	–
	2	16	8	3	3	6	6	3	1	0	1	0 (+ 0) / 1 (+ 0)	1 (+ 0)^g^
*6430573F11Rik*	1	9	5	0	2	3	3	0 (+ 1)	–	– (+ 1)	–	– (+ 0) / – (+ 1)	– (+ 1)
	2	21	14	2^e^	1	7	6	2^e^	0	–	2	–	–
	3	28	3	0	1	3	2	0	–	–	–	–	–
	4	5	3	0	1	1	3	0	–	–	–	–	–
*Acvr2b*	1	11	8	0	1	0	7	0	–	–	–	–	–
*Inpp5k*	1	4	2	0	1	0	1	0	–	–	–	–	–
	2	21	11	2	2	6	8	2	0	0 (+ 0)	2	–	–

The table summarizes the numbers of animals involved in the generation of conditional alleles employing CRISPR/Cas9 reagents and lssDNA donors and the outcome of mutagenesis. The table also shows the outcome of the breeding of positive founders, each generating a new line. Further summaries and sequencing data for each of the projects detailed in Table 1 are shown in Additional file 2: Figure S1, Additional file 3: Figure S2, Additional file 4: Figure S3, Additional file 5: Figure S4, Additional file 6: Figure S5, Additional file 7: Figure S6, Additional file 8: Figure S7, Additional file 9: Figure S8, Additional file 10: Figure S9 and Additional file 11: Figure S10

^a^*MS* microinjection session

^b^Founders with progeny for transmission of floxed allele (in brackets founders bred for transmission of deletion allele only)

^c^One additional deletion allele identified by sequencing

^d^Founder with floxed allele died before mating

^e^One founder with floxed allele with point mutation in intron found in F_0_

^f^Random insertion of donor sequence detected by ddPCR in line but not transmitted to all F_1_ animals of interest, e.g. cKO/exon deletion

^g^Random insertion detected but not associated with allele of interest, e.g. cKO/exon deletion

#### Screening of F_0_ generation and genotyping of F_1_ animals

As animals of the F_0_ generation were likely to be mosaic, we analyzed them by screening for the presence of the allele of interest [13]. Polymerase chain reaction (PCR) amplicons were produced from genomic DNA with primers flanking the homology arms and external to the donor (Syt7 primers R1 and F1, Fig. 1a). Their analysis on agarose showed two founders (Fig. 1b, Animals Syt7-1 and Syt7-6) containing deletions. The PCR products from founder animals were purified and sequenced by Sanger sequencing. The sequencing showed that a total of 10 animals out of 17 were mutated on target (*Syt7*, Table 1). Among them, five pups had indels at either or both 5′ and 3′ guide target sites. Three other animals (Syt7-1, Syt7-6 and Syt7-9) carried alleles with deletions of the sequence flanked by the two pairs of sgRNAs corresponding to non-cKO alleles. The remaining two mutants (Syt7-4 and Syt7-8) were carriers of the designed cKO allele, with sequencing traces suggesting Syt7-8 to be homozygous and Syt7-4 compound heterozygous with one cKO allele and one allele including the 3′ loxP and an indel in 5′ (Additional file 2: Figure S1).

Positive founders Syt7-4 and Syt7-8 were mated to wild-type (WT) animals, and the progeny (F_1_) were analyzed. In contrast to the analysis of mosaic F_0_ animals, sequencing of PCR fragments amplified from F_1_ individuals allowed for definitive characterization of the edited alleles [13]. The outcome of the analysis of F_1_ animals by PCR and sequencing, employing the same primers used for screening F_0_ animals, is summarized in Table 2. Sequencing showed successful transmission of the correctly mutated sequence (cKO allele) by both founders to their progeny (individuals Syt7-4.1d and Syt7-8.1c, e, f and g).

**Table 2:** Characterization of animals for the generation of a *Syt7* conditional allele

Founder ID	Allele type 1	Allele type 2	Allele type 3	Copy number	F_1_ animal ID	PCR and sequencing outcome	Copy number	Allele 1	Allele 2
Syt7-4	cKO	5’ NHEJ + 3’ loxP	Deletion^c^	1.03 ± 0.07	4.1a	Only WT allele amplified	1.08 ± 0.04	WT	Deletion^c^
					4.1b	Only WT allele amplified	1.02 ± 0.04	WT	Deletion^c^
					4.1c	Only WT allele amplified	1.09 ± 0.06	WT	Deletion^c^
					4.1d	Both loxP present	1.99 ± 0.08	WT	cKO
Syt7-8	cKO	Appears homozygous^a^	Additional insertion^b^	2.78 ± 0.15	8.1a	Only WT allele amplified	1.00 ± 0.07	WT	Deletion^c^
					8.1b	Only WT allele amplified	1.93 ± 0.08	WT	WT
					8.1c	Both loxP present	2.98 ± 0.09	WT	cKO + Additional insertion^b^
					8.1d	Only WT allele amplified	2.93 ± 0.09	WT	WT + Additional insertion^b^
					8.1e	Both loxP present	2.07 ± 0.07	WT	cKO
					8.1f	Both loxP present	2.13 ± 0.07	WT	cKO
					8.1g	Both loxP present	2.89 ± 0.15	WT	cKO + Additional insertion^b^
					8.1h	Only WT allele amplified	2.90 ± 0.15	WT	WT + Additional insertion^b^

The table summarizes the results of screening of the F_0_ animals obtained for the generation of a conditional *Syt7* allele and of the F_1_ animals produced from the mating of F_0_ positive animals to WT mice. Outcomes of PCR and Sanger sequencing characterization employing the Syt7-F1 and Syt-R1 primers external to the lssDNA donor and copy counting of the donor, where relevant, are shown. Sequencing data showing a correct conditional allele is shown in Additional file 3: Figure S2a

^a^Second legitimate repair or combined with large deletion, unclear at F_0_ stage

^b^Revealed by copy number, on or off target

^c^Deletion including at least one external genotyping primer site

Screening of mutants obtained by co-injection of transcription activator-like effector nuclease (TALEN) and ssODNs showed that random integration of ssODNs can occur when using such a mutagenesis approach [20], illustrating the requirement of further validation of positive animals by a method allowing copy counting. We therefore checked for the presence of additional copies of the lssDNA donor sequence in the genome of F_0_ and F_1_ animals using digital droplet PCR (ddPCR) and a TaqMan™ assay centred on the critical exon present in the donor sequence run against a known two-copy reference assay (*Syt7* exon 7, *Dot1l* reference assay, as per [13]). Table 2 shows the copy number of the donor sequence in each individual, illustrating the presence of additional copies in some F_0_ (Syt7-8) and F_1_ individuals (Syt7-8.1c, d, g and h).

In particular, copy counting for founder Syt7-8 (which was suggested as a potential homozygous for the cKO allele by PCR and sequencing) also revealed additional integrations of the lssDNA donor (close to 2.8 copies per genome, Table 2). The copy number obtained in the founder is not a clear integer number, which is not impossible in a mosaic animal. Analysis of the F_1_ progeny confirmed the presence of an additional integration (Syt7-8.1c, d, g and h) and strongly suggested that this event was not physically linked to the targeted allele in the founder, as this integration could be segregated from the mutated allele in other F_1_ progeny (Syt7-8.1e and f).

Copy counting of the critical exon also confirmed deletions of the target region in some F_0_ (Syt7-4) and F_1_ individuals (Syt7-4.1a, b and c; Syt7-8.1a). The ddPCR analysis also showed a reduced copy number of exon 7 in F_1_ animals initially thought to be WT as an exon deletion had not been detected by standard PCR with external primers (Syt7-4.1a, b and c; Syt7-8.1a) Table 2. This suggests that these animals were bearing a deletion larger than the segments flanked by the genotyping primers.

In summary, the delivery of lssDNA donor together with CRISPR/Cas9 reagent to a modest number of one-cell embryos produced mosaic animals that transmitted a conditional allele. Some of the transmitting progeny were excluded upon further validation steps due to additional integrations of donor sequence.

### Other conditional alleles

#### Production of F_0_ animals

The pilot was next extended to include a further eight genes with the same design principles (Table 1 and Additional file 1: Table S2): Two sgRNAs were selected on each side of a critical exon in the genomic sequences to be interrupted by the loxP sites (details of sequences are given in Additional file 1: Table S1, designs in Additional file 4: Figure S3). Refining our strategy in the process of extending the pilot, we introduced standard sequences flanking the loxP sites in the designs, thus allowing us to re-use established diagnostic tests for the validation of alleles (restriction enzyme sites or LoxP-F and LoxP-R primers in Additional file 4: Figure S3). This facilitated the analysis of animals. CRISPR/Cas9 reagents and lssDNA were delivered to C57BL/6NTac one-cell embryos by pronuclear injection.

#### Screening of F_0_ generation and genotyping of F_1_ animals

F_0_ and F_1_ animals were analyzed according to the same strategy as that used for the *Syt7* conditional allele: PCR using primers external to the donor homology arms (or two PCRs bridging the homology arms, depending on PCR efficiency) and a PCR amplifying the region flanked by the two loxP sites, all of which were analyzed by Sanger sequencing (Additional file 5: Figure S4, Additional file 6: Figure S5, Additional file 7: Figure S6, Additional file 8: Figure S7, Additional file 9: Figure S8, Additional file 10: Figure S9, Additional file 11: Figure S10 and Additional file 12: Figure S11). A total of 279 F_0_ animals were analyzed, and 129 animals were identified as bearing mutations. Seven out of nine projects yielded founders bearing the conditional allele, with an additional one yielding a floxed allele with an unwanted point mutation. One project (Rapgef5) only yielded one founder bearing a conditional allele, that died before mating age. Correct conditional alleles were transmitted to the F_1_ generation for four out of the seven projects where founder progeny were analyzed (Table 1). However, in at least three out of nine projects, other alleles were detected which contained unexpected point mutations identified at the F_0_ generation (Inpp5k project, Additional file 12: Figure S11h; 6430573F11Rik project, Additional file 13: Figure S12a; Cx3cl1 project, Additional file 13: Figure S12b and c).

It is also noteworthy that illegitimate repairs [7] or partial integration(s) of the donor were detected frequently (in eight out of nine projects analyzed, see example in (Additional file 12: Figure S11d), highlighting the requirement of extensive allele validation by PCR and sequencing. These events—point mutations, partial and/or rearranged integrations—are reported as illegitimate repairs in Table 1.

Interestingly, F_0_ animals with exon deletions were generated in all but one project as a by-product. Whenever null animals were required for ongoing research, these founders were also mated (numbers in brackets, Table 1). So far, germline transmission (GLT) of this additional allele was obtained in five out of six projects where positive founders were bred.

It is noteworthy that two out of these nine projects (Ikzf2 and Usp45) had been previously attempted employing ssODNs or plasmids without yielding founders with conditional alleles, in contrast to subsequent attempts with lssDNA donors (Additional file 1: Table S3).

F_0_ and F_1_ animals containing the cKO alleles were further validated by copy counting with a TaqMan™ assay centred on the floxed region. Importantly, copy counting of the floxed region in combination with the outcome of the targeted allele validation showed additional integrations in four out of seven projects analyzed (Table 1).

### Point mutations remote from active sgRNA cutting site

#### Production of F_0_ animals

**Fig. 2:**
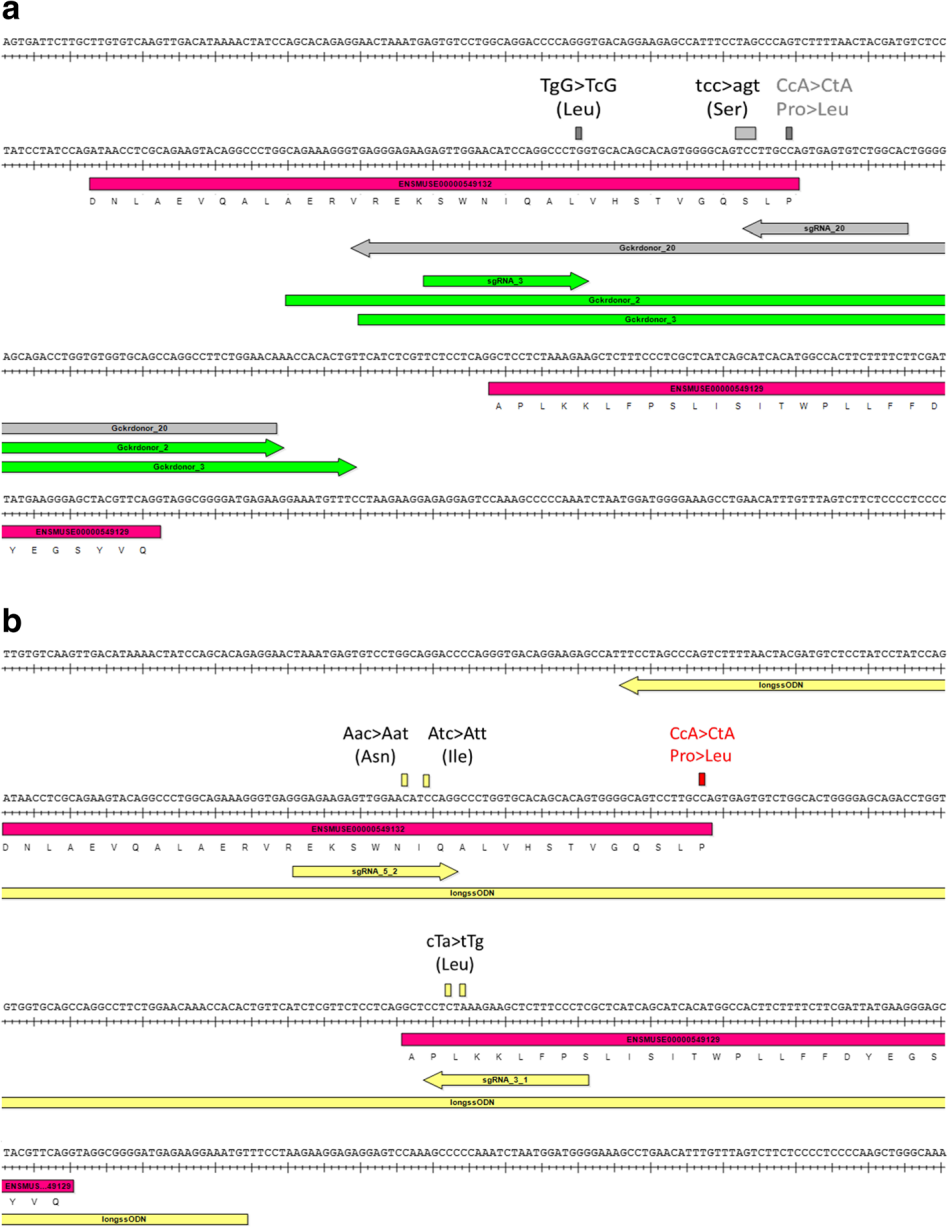
Gckr^P446L^ point mutation. Different designs of reagents for genome editing employing (**a**) oligonucleotides or (**b**) a lssDNA donor. Donors were designed containing both coding (in red) and silent mutations (in black) that prevent reprocessing of engineered alleles in accordance with the selected sgRNAs. Guide sequences are named sgRNAs. The shared colour coding of guides and donors highlights reagents injected within the same mix

**Fig. 3:**
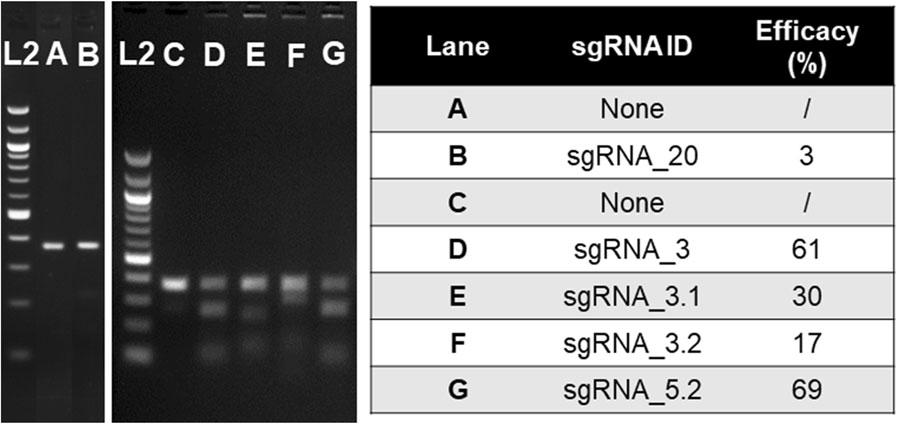
Guide-it validation of the five sgRNAs synthesized for the generation of the Gckr^P446L^ point mutation. Cas9 protein is complexed with each sgRNA (B, D–G) and incubated with short double-stranded DNA fragments containing the protospacer target. Lanes A and C are controls and show the target template but no Cas9/sgRNA complex. The reactions are analyzed for cleavage by electrophoresis on agarose gel. L2 = 100 bp DNA molecular weight ladder (thick bands are 1000 and 500 bp). Protospacer sequences are detailed in Additional file 1: Table S1

Finally, we assessed whether the production of a point mutation distal from an active sgRNA cutting site, the generation of which has so far been unsuccessful by repeated attempts using other methods, could also be facilitated by the use of lssDNA. The first target for this pilot was the generation of the Gckr^P446L^ point mutation in C57BL/6NTac mouse embryos (sequence change illustrated in Additional file 15: Figure S14). We initially designed a strategy according to the standard approach, employing a ssODN and one efficient and specific sgRNA cutting as close as possible to the targeted nucleotide. However, some factors limited options for design, such as the close proximity of the target to the exon-intron junction and splice sites that should not be altered. Furthermore, the poor specificity of the target sequence (sequence conserved and repeated at two additional locations in the mouse genome; GRCm38.p5:10:82265447–82265469/12:21568953–21568975) rendered many guides unspecific. The closest sgRNA to the target nucleotide (sgRNA_20 (Fig. 2a)) was shown to be inactive by a Guide-it™ assay, where the CRISPR/Cas9 nuclease activity is assessed on a target DNA fragment in vitro (Fig. 3). This was subsequently confirmed by the fact that no mutagenesis was detected in microinjection session 1 where this sgRNA was used. Therefore, the closest efficient (as confirmed by Guide-it™ assay) and specific sgRNA that could be selected was cutting 34 nt away from the targeted base pair (sgRNA_3, Figs. 2a and 3). Thus, our next strategy employed sgRNA_3 and a ssODN donor, although a distance larger than 30 bp between the target sequence and the cutting site of the sgRNA can represent a barrier to the generation of a specific point mutation [9]. In addition to the targeted nucleotide mutation, a silent mutation was included in the ssODN donor template in order to abolish the protospacer adjacent motif (PAM) of the selected sgRNA and prevent re-processing of the mutated allele by the CRISPR/Cas9 system (Fig. 2a). The sgRNA activities were checked in vitro (Fig. 3), and each RNA was co-injected with Cas9 mRNA and the ssODN, as per the designs shown in Fig. 2a and Additional file 1: Table S1.

**Fig. 4:**
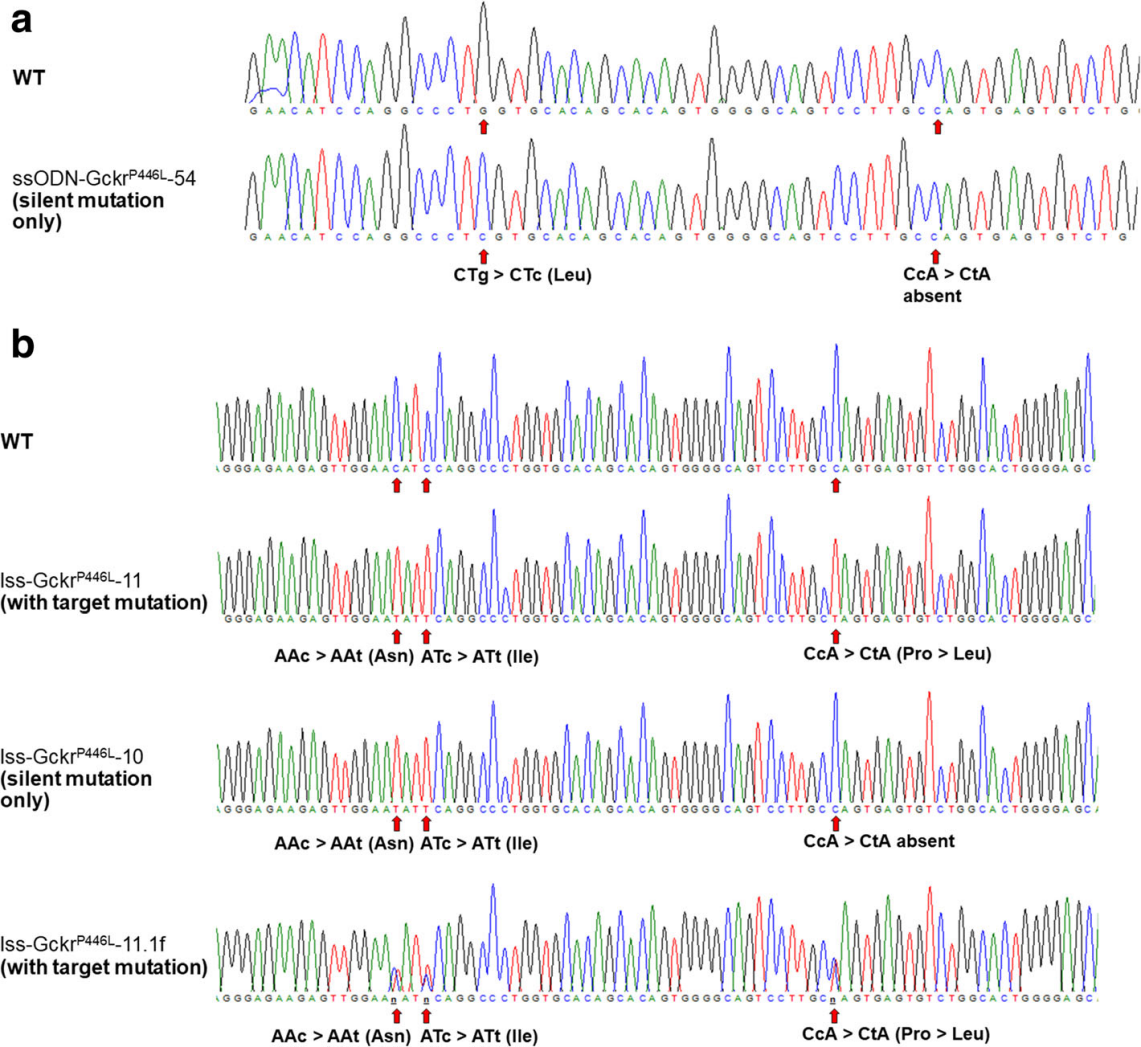
Screening by Sanger sequencing of animals for the generation of the Gckr^P446L^ point mutation with (**a**) oligonucleotides (F_0_ individual ssO-Gckr^P446L^-54) or (**b**) lssDNA donors (F_0_ individuals lss-Gckr^P446L^-11 and lss-Gckr^P446L^-10 and F_1_ individual lss-Gckr^P446L^-11.1f). The figure shows Sanger sequencing chromatograms of an amplicon generated with primers anchored external to the intended site of donor sequence integration as detailed in Additional file 15: Figure S14. **a** ssODN donors only yielded introduction of the intended silent mutations, while (**b**) lssDNA yielded the desired mutation in some individuals (F_0_ 11 transmitting to 11.f) and only the silent mutations in others (F_0_ 10). Note that founders appeared homozygous (ssO-Gckr^P446L^-54, lss-Gckr^P446L^-11 and lss-Gckr^P446L^-10) when analyzed by Sanger sequencing, but also could contain deletion alleles in *trans*, as suggested by copy counting (lss-Gckr^P446L^-11 in Table 4). A summary of the microinjection session outcomes is detailed in Table 3, and raw sequencing data are provided in Additional file 16

We anticipated that generating the desired mutation would be challenging, as the target base is a sub-optimal 34 base pairs away from sgRNA_3’s cut site. We therefore performed multiple injection sessions with two different ssODN designs (Gckrdonor_2 and Gckrdonor_3, centred or offset towards the targeted mutation, respectively; sequences in Additional file 1: Table S1) to enhance the likelihood of obtaining the desired point mutation. The outcome of these microinjections was analyzed by PCR and sequencing of the region of interest in a total of 90 pups and is summarized in Table 3. Although the silent mutation was detected in F_0_ animals on five occasions, it was not accompanied by the mutation of interest (Table 3 and example in Fig. 4a, ssO-Gckr^P446L^-54). Sequencing data from founders are shown in Additional file 16.

**Table 3:** Generation of a Gckr^P446L^ point mutation

						F_0_ with:					
MS	Donor type	Guide ID(s)	Donor ID	Embryos transferred	F_0_ biopsied (birth rate)	Mutation	Correct mutation	SM only	SM only and rearranged	NHEJ alleles	Random integration
1	ssODN	20	Gckrdonor_20	80	12 (15%)	0	0	0	0	0	n.d.
2	ssODN	3	Gckrdonor_2	80	21 (26%)	12	0	2	2	9	n.d.
3	ssODN	3	Gckrdonor_2	70	13 (19%)	4	0	0	0	4	n.d.
4	ssODN	3	Gckrdonor_2	135	18 (13%)	9	0	2	1	7	n.d.
5	ssODN	3	Gckrdonor_2	42	10 (23%)	3	0	0	0	3	n.d.
6	ssODN	3	Gckrdonor_3	121	8 (7%)	5	0	1	1	3	n.d.
7	ssODN	3	Gckrdonor_3	112	8 (7%)	3	0	0	0	3	n.d.
1	lssDNA	5.2, 3.1	Gckr_P446L_lss	210	22 (10%)	14	8	1	2	7	0/2

The table shows the numbers of embryos and animals involved in mutagenesis attempts employing the injection of CRISPR/Cas9 reagents and oligonucleotides or lssDNA donors. The percentage of transferred embryos yielding live animals at weaning is shown in parentheses. The outcome of these attempts is also summarized. Note that sgRNA_20 was employed for the first microinjection session with ssODN_20 and substituted to sgRNA_3 and relevant donor ssODNs for subsequent sessions, as it was confirmed to be inactive. Sequencing data from this project are displayed in Fig. 4 (additional raw sequencing data are provided in Additional file 16)

*MS* microinjection session, *n.d.* not determined *SM* silent mutation

We subsequently designed an alternative strategy employing a larger (339 bases) lssDNA sequence and two sgRNAs flanking the region containing the targeted nucleotide. The sgRNAs were selected to introduce double-stranded breaks on each side of the target (40 and 98 nt away in 5′ and 3′, respectively), and their activity was checked in vitro. We consequently selected sgRNA_5.2 and sgRNA_3.1 as they were shown to be most active in vitro (Figs. 2b and 3). The donor sequence was designed with 100 nt homology arms flanking the cut sites, silent mutations that modify the seed sequences of the selected sgRNAs to prevent re-processing and the targeted base change (Fig. 2b). The lssDNA was synthesized in accordance with prior experiments and co-injected with Cas9 mRNA and the two sgRNAs in a single session, the outcome of which is shown in Table 3. Twenty-two pups were weaned, and ear biopsies were taken to screen for new alleles.

#### Screening of F_0_ generation and genotyping of F_1_ animals

Primers were designed in genomic regions flanking, but external to, the donor sequence to span the donor integration (Gckr^P446L^-F2 and Gckr^P446L^-R2 primers, Additional file 1: Table S1 and Fig. 2b). PCR amplicons were synthesized from genomic DNA and sequenced by Sanger sequencing. Sequencing data from all founders are shown in Additional file 16.

Sequencing showed that 14 animals out of 22 were mutated on target. Among them, eight individuals carried the designed knock-in (KI) allele (Table 3), with sequencing traces suggesting that four animals were homozygous for the KI (Fig. 4b). Three other individuals showed illegitimately repaired alleles (Table 3 and silent mutation only Fig. 4b).

Two of the four apparently homozygous positive F_0_s (lss-Gckr^P446L^-11, lss-Gckr^P446L^-19) were mated to WT animals for GLT of the mutated allele. The analysis of F_1_ animals (summarized in Table 4) showed the successful transmission of the correctly mutated sequence by both founders (i.e. lss-Gckr^P446L^-11.1f, Fig. 4b).

**Table 4:** Analysis of the Gckr^P446L^ project

Founder ID	Allele 1	Allele 2	Allele 3	Copy number	F1 animal ID	PCR and sequencing outcome	Copy number	Allele 1	Allele 2
lss-GckrP446L-11	Legitimate repair	Legitimate repair	Deletion^b^	1.47 ± 0.11	11.1a	WT	1.53 ± 0.07	WT	WT?^a^
					11.1b	WT	1.03 ± 0.04	WT	Deletion
					11.1c	Legitimate repair and WT	1.88 ± 0.10	WT	Legitimate repair
					11.1d	WT	1.05 ± 0.06	WT	Deletion
					11.1e	WT	1.01 ± 0.05	WT	Deletion
					11.1f	Legitimate repair and WT	1.85 ± 0.06	WT	Legitimate repair
					11.1g	Legitimate repair and WT	1.85 ± 0.12	WT	Legitimate repair
					11.1h	WT	1.01 ± 0.05	WT	Deletion
lss-GckrP446L-19	Legitimate repair	Legitimate repair	Deletion^b^	1.44 ± 0.18	19.1a	Legitimate repair and WT	1.90 ± 0.09	WT	Legitimate repair
					19.1b	Legitimate repair and WT	1.81 ± 0.12	WT	Legitimate repair

The table details the results of screening of two positive F_0_ animals obtained for the generation of a Gckr^P446L^ point mutation and the subsequent characterization of the F_1_ animals obtained from mating of these F_0_ animals to WT mice

^a^Deletion affecting the region recognized by the TaqMan™ assay

^b^Revealed by copy number

#### Further model validation

We also checked for the presence of additional copies of the donor sequence in the genome of F_0_ and F_1_ animals using ddPCR and a TaqMan™ assay centred on the donor sequence (as per [13]). Table 4 shows the copy number of the donor sequence in each individual, illustrating a deletion likely spanning a fragment larger than the segments flanked by the genotyping primers (individuals lss-Gckr^P446L^-11.1a, b, d, e and h, Table 4). Although both founders appeared homozygous for the point mutation by Sanger sequencing, lss-Gckr^P446L^-11 also transmitted a deletion allele to its progeny, confirming mosaicism in this individual.

We next attempted to employ lssDNA donors for the generation of a mouse line bearing a point mutation in the *Rims1* gene, which also had not been achieved with standard ssODN donors (Additional file 17: Figure S15 and Additional file 18: Figure S16; Additional file 1: Table S4, 1 positive founder/155 animals born (0.6%); this founder did not yield GLT, Additional file 1: Table S5). The new design employing lssDNA (Additional file 17: Figure S15) yielded founders bearing the correct mutation at a much higher frequency (4 positive founders/39 animals born (10%) with lssDNA donors), one of which achieved GLT of this second challenging point mutation (Additional file 1: Tables S4 and S5; Additional file 19: Figure S17; sequencing data in Additional file 20). Sequencing data from all founders for the point mutation (with ssODNs and lssDNA donors) are shown in Additional file 20.

## Discussion

### Novel strategy for challenging point mutations

Standard methods employing chemically synthesized oligonucleotides had not permitted the introduction of the Gckr^P446L^ point mutation (Table 3), although evidence of partial integration of the donor (silent mutation) was recorded in five animals. This is likely due to the distance between the available sgRNA and the target sequence (34 bp). We have extended the pilot to a second challenging point mutation and also found that the use of a lssDNA donor yielded the generation and GLT of the point mutation (Additional file 1: Tables S4 and S5; Additional file 19: Figure S17), reinforcing the proposition that the use of lssDNA can rescue such unsuccessful projects. This study is the first proof of principle that the use of lssDNAs can lift the barrier to the introduction of hitherto challenging point mutations into the mouse genome, where no active and/or specific sgRNA is available in the immediate vicinity of the target site. Extending our capacity to generate point mutations further away from available optimal sgRNA target sites is of crucial importance, as it will enable the generation of thus far challenging mutants, including those models essential for the validation of candidate mutations causing human disease arising from whole genome sequencing (WGS) or quantitative trait locus (QTL) analysis [21].

### Alternative methods for production of lssDNA donor

We chose IVT followed by reverse transcription as a method to obtain lssDNAs [10]. Alternative methods employing combined nickase and nuclease digestion of a plasmid [22], use of a biotin-labelled primer [23], conversion of double-stranded DNA to ssDNA by nucleases (Guide-it™ Long ssDNA Production System, Takara) or chemical synthesis [11] have been proposed. However, synthesizing lssDNA donor molecules remains a challenge: the IVT-based method is both lengthy and expensive; the use of nucleases can give limited yield and requires DNA of impeccable quality; and chemical synthesis is expensive and also has size limitations. It will be important to refine or replace these methods to facilitate access to high-quality donors.

### Efficiency of model generation

Many advancements in the rapidly evolving genome editing field have been published on the basis of a small number of experiments, and these have sometimes proven to be difficult to reproduce [24, 25]. Our results support the view that lssDNAs facilitate the production of complex alleles, suggesting that the method as described by Quadros and colleagues [11] is sufficiently robust for reproducibility between laboratories.

Two of these projects (Ikzf2 and Usp45) were initially attempted employing ssODNs or plasmids as donors, but only the switch to lssDNA has yielded founders with conditional alleles, suggesting it is a more successful method (previous approaches and their outcomes are summarized in Additional file 1: Table S3). We note that other labs have encountered some successes with ssODN donors and otherwise very similar methods for the generation of cKOs ([3], this issue, Lanza et al. [18]). However, the use of lssDNA as donors has proven more efficient in our hands than that of ssODNs, when compared for the generation of the same mutations (*Ikzf2* conditional allele and *Gckr* and *Rims1* point mutations). In particular, it alleviates the challenge of integrating both loxP sites in the same allele when generating cKOs and facilitates the introduction of point mutations away from active sgRNA active sites.

It is not yet clear why lssDNAs are proving to be superior donor molecules in this context, but their particular efficiency is likely not due to the length of homology arms used in lssDNA donors (up to 100 bases), as much larger homologous sequences were present in plasmid donors.

However, not all projects were successful. The efficiency of this method is likely to be reliant on sufficiently active sgRNAs on both sides of the sequence to be integrated (i.e. the Acvr2b project did not yield conditional alleles or any deletions). It is therefore prudent to check the activity of sgRNAs in vitro and design the donor sequence according to which sgRNAs are the most active. Also, GLT of the floxed allele relies on the viability and fertility of mosaic founders, as illustrated by the failure so far of the Rapgef5 project to yield a conditional allele. Finally, some failures were due to unwanted single nucleotide changes (examples in Additional file 13: Figure S12), most likely picked up during the lssDNA generation process. It is our prediction that some of these failures, but not all, will be reversed by further repeat attempts.

In summary, our data support efficiency, but not all models were achieved. Interestingly, the process also produced exon deletion alleles as a by-product of the generation of cKOs, allowing rapid access to null alleles.

### Mutant validation

Mutant validation was performed by PCR, employing genomic primers external to the donor sequence and systematic sequencing of the integration, as well as copy counting of the donor sequence.

#### Validation of mutated allele

We and others have previously described that imperfect alleles can be generated when using ssODNs as donors (“illegitimate repairs” [7], “KI + indels” [9]). Further, rearranged alleles have also been detected when no donor is included in the mutagenesis strategy [7, 12, 26]. Here we show that rearrangements also occur in the presence of lssDNA donors (Table 1 and example in Additional file 14: Figure S13). As such, the use of lssDNA does not lessen the requirement for allele validation by full sequencing, as rearrangements (including indels and partial integrations) may occur during the double-strand break repair event. In addition, the synthesis of lssDNA itself can be a source of errors [27], potentially introducing unwanted sequence changes early in the process that will require monitoring by full sequencing of the allele. The use of new high-fidelity enzymes (including a replacement of standard reverse transcriptase) might contribute to reducing the frequency of sequence errors in the edited alleles.

Inclusion in the donor of sequences of known primers that are specific and efficient in PCR or restriction enzyme sites can simplify screening for mutated loci but does not replace QC by sequencing. Alternative methods for validation of new alleles, involving string sequencing for example, could further facilitate QC.

#### Additional integrations

Our results show that additional donor integrations are common (five out of six projects; this was also found in [18]). Even when there is no evidence of such an event in the founder generation, it is essential to check for their presence at the F_1_ stage, as there is a clonal event at the point of GLT. Furthermore, if the mutant-specific genotyping assay used in subsequent generations is internal to the donor sequence, it will not discriminate between on-target and unidentified additional integrations. Copy counting can be performed by quantitative PCR (qPCR) or most easily by ddPCR, employing an assay centred on the donor that will recognize both WT and mutant alleles (universal) or a mutation-specific assay in correlation with sequencing of a locus-specific amplicon (amplified with primers external to the donor). The locations of random integrations were not identified, so it is unclear whether they were associated with CRISPR/Cas9 off-target activity.

#### Standards for quality control

We found examples of sequence changes, indels, locus rearrangements or random insertion of lssDNA donors in all projects attempted, showing that mutagenesis artefacts are very common. Full model validation at the F_1_ stage is therefore essential, and it constitutes a labor-intensive exercise involving the sequencing of large or several overlapping amplicons and copy counting of donor insertions. The need for extensive model validation is not specific to the use of lssDNA in genome editing [9, 13, 20], but it is not alleviated by the use of this new donor type.

Publications reporting proof-of-principle cases for using the CRISPR/Cas9 system for genome engineering focus on the novelty of methods and often do not include the intricacies of QC of mutants [2, 3, 11]. However, thorough validation of new models is essential to the reproducibility of research employing mutated laboratory animals. This can be a complex exercise, as genome editing can yield many unpredicted events, both on-target and in other loci. There are profound consequences in using mouse lines harbouring additional mutations in ongoing research, including misleading results, erroneous interpretations of study and avoidable animal wastage. Therefore, the dissemination of good practice for QC is just as essential as the distribution of efficient protocols for mutagenesis. Also, an extensive validation of mouse mutants is indispensable to providing a complete documentation of animals used in research [14].

## Conclusion

Prior to the use of lssDNA, the reliable generation of complex alleles and some point mutations remote from efficacious sgRNA target sequences was out of reach. Here, we have shown the application of lssDNA to both the generation of cKO alleles and challenging point mutations. However, the technique can also produce a variety of artefacts: point mutations, indels, locus rearrangements and additional donor integrations. A comprehensive mutant validation strategy involving sequencing of the locus and copy counting of the donor is therefore essential. The utilization of lssDNA as a donor sequence lifts the barrier to the generation of complex alleles and shifts the challenge of the exercise from the production of founders bearing these new alleles towards the validation of these new mutants.

## Methods

### sgRNAs

Guide sequence selection was carried out using the following online tools: CRISPOR [28] and Wellcome Trust Sanger Institute (WTSI) Genome Editing (WGE) [29]. sgRNA sequences were selected with as few predicted off-target events as possible, particularly on the same chromosome as the intended modification. sgRNAs used in this study are shown in Additional file 1: Table S1. sgRNAs were synthesized directly from gBlock® (IDT, Skokie, IL, USA) templates containing the T7 promoter using the HiScribe™ T7 High Yield RNA Synthesis Kit (New England BioLabs®, Ipswich, MA, USA) following manufacturer’s instructions. RNAs were purified using the MEGAclear Kit (Ambion). RNA quality was assessed using a NanoDrop spectrophotometer (ThermoScientific) and by electrophoresis on 2% agarose gel containing ethidium bromide (Fisher Scientific). A Guide-it™ assay was performed as per manufacturer instructions (Takara, Kyoto, Japan).

### Templates for lssDNA synthesis

Templates for lssDNA synthesis were either assembled by cloning in a plasmid or, when possible, were obtained from IDT as a single gBlock®. Additional file 1: Table S1 details the generation of the lssDNA employed in this study.

### Donor sequences

Donor ssODNs (desalted grade) were obtained from IDT. Donor lssDNAs were initially generated following a method adapted from [10]. Briefly, templates for IVT (donor sequence flanked by the T7 promoter) were obtained as a gBlock® (IDT) or cloned in a plasmid that was subsequently linearized. Typically, 150 ng of double-stranded gBlock® template or 2 μg of plasmid template was transcribed using the HiScribe T7 High Yield RNA Synthesis Kit (New England BioLabs®). At the end of the reaction, DNase I was added to remove the DNA template. RNA was purified employing the MEGAclear Transcription Clean-Up Kit (Ambion). Single-stranded DNA was synthesized by reverse transcription from 20 μg of RNA template employing SuperScript III Reverse Transcriptase (Invitrogen), treated with RNAse H (Ambion) and purified employing the QIAquick Gel Extraction Kit (Qiagen, Hilden, Germany). Donor concentration was quantified using the NanoDrop (Thermo Scientific), and the integrity was checked on 1.5% agarose gel containing ethidium bromide (Fisher Scientific).

### Mixes for microinjection

Microinjection buffer (10 mM Tris-HCl, 0.1 mM ethylenediaminetetraacetic acid (EDTA), 100 mM NaCl, pH 7.5) was prepared and filtered through a 2-nm filter and autoclaved. Mixes containing 100 ng/μl Cas9 mRNA (5meC,Ψ) (TriLink BioTechnologies, San Diego, CA, USA), 50 ng/μl gRNAs and 50 ng/μl ssODN or 50 ng/μl lssDNA were prepared in microinjection buffer, filtered through Costar® SpinX® Centrifuge Tube Filters (Corning) and stored at − 80 °C until microinjection.

### Mice

All animals were housed and maintained in the Mary Lyon Centre, MRC Harwell Institute under specific-pathogen-free (SPF) conditions, in individually ventilated cages adhering to environmental conditions as outlined in the Home Office Code of Practice. Mice were euthanized by Home Office Schedule 1 methods. Colonies established during the course of this study are available for distribution and are detailed in Additional file 1: Table S6.

### Pronuclear microinjection of zygotes

All embryos were obtained by superovulation. Pronuclear microinjection was performed as per Gardiner and Teboul [30], employing a FemtoJet (Eppendorf AG, Hamburg, Germany) and C57BL/6NTac embryos for all projects shown here, apart from Rims1, which was performed with C57BL/6J embryos. Specifically, the injection pressure (*P*_i_) was set between 100 and 700 hPa, depending on the needle opening; the injection time (*T*_i_) was set at 0.5 s and the compensation pressure (*P*_c_) was set at 10 hPa. Mixes were centrifuged at high speed for a further minute prior to microinjection. Injected embryos were re-implanted in CD-1 pseudopregnant females. Host females were allowed to litter and rear F_0_s.

### Breeding for germline transmission

F_0_ animals where the presence of a desired allele was detected were mated to WT isogenic animals to obtain F_1_ animals to assess the GLT of the allele of interest and permit the definitive validation of its integrity.

### Genomic DNA extraction ear biopsies

Genomic DNA from F_0_ and F_1_ animals was extracted from ear clip biopsies using the DNA Extract All Reagents Kit (Applied Biosystems) according to the manufacturer’s instructions. The crude lysate was stored at − 20 °C.

### PCR amplification and sequencing

New primer pairs were set up in a PCR reaction containing 500 ng genomic DNA extracted from a WT mouse, 1× Expand Long Range Buffer with 12.5 mM MgCl_2_ (Roche), 500 μM PCR Nucleotide Mix (dATP, dCTP, dGTP, dTTP at 10 mM, Roche), 0.3 μM of each primer, 3% dimethyl sulfoxide (DMSO) and 1.8 U Expand Long Range Enzyme mix (Roche) in a total volume of 25 μl. Using a T100 thermocycler (Bio-Rad, Hercules, CA, USA), PCRs were subjected to the following thermal conditions: 92 °C for 2 min followed by 40 cycles of 92 °C for 10 s, a gradient of annealing temperatures between 55 and 65 °C for 15 s and 68 °C for 1 min/kilobase and a final elongation step for 10 min at 68 °C. The PCR outcome was analyzed on a 1.5–2% agarose gel, depending on the amplicon size, and the highest efficient annealing temperature was identified for the primer pair. If no temperature allowed for an efficient and/or specific PCR amplification, the assay was repeated with an increased DMSO concentration (up to 12%). Using optimized conditions as defined above, PCRs for each project were run and an aliquot analyzed on agarose gel. The PCR products were purified employing a QIAquick Gel Extraction Kit (Qiagen) and sent for Sanger sequencing (Source Bioscience, Oxford, UK). Genotyping primers were chosen to be at least 200 bp away from the extremity of donors, depending on available sequences for design.

### Sequencing data analysis

Sequencing data were analyzed differently depending on whether they were obtained from F_0_s or F_1_s (as per [13]). At the F_0_ stage, animals were screened for evidence of the expected change, i.e. the presence of loxP sites for conditional allele projects or the presence of the expected base change for the Gckr^P446L^ point mutation project. F_0_ animals should be considered mosaic animals. All F_1_ animals are heterozygous containing one WT allele and one allele to be determined, as they are obtained from mating F_0_ animals with desired gene edits to WT animals. The F_1_ stage enables definitive characterization of the new mutant.

### Sub-cloning of PCR products

PCR products amplified from F_0_ DNA showing complex sequencing traces were sub-cloned using a Zero-Blunt PCR Cloning Kit (Invitrogen). The appropriate number of clones (usually 12–24) per founder were picked and grown overnight in accordance with the complexity of the traces observed prior to sub-cloning. Plasmids were isolated using a QIAprep Miniprep Kit (Qiagen) and analyzed by Sanger sequencing (Source Bioscience) using the M13R oligonucleotide or gene-specific primers.

### ddPCR

Copy number variation experiments were performed as duplex reactions, where the sequence employed as a donor was amplified using a fluorescein amidite (FAM)-labelled assay (sourced from Biosearch Technologies, Petaluma, CA, USA), in parallel with a VIC-labelled reference gene assay (*Dot1l*, sourced from ThermoFisher) set at two copies (CNV2) on the Bio-Rad QX200 ddPCR System (Bio-Rad) as per Codner and colleagues [31]. Reaction mixes (22 μl) contained 2 μl crude DNA lysate or 50 ng of phenol/chloroform purified genomic DNA, 1× ddPCR Supermix for probes (Bio-Rad), 225 nM of each primer (two primers per assay) and 50 nM of each probe (one VIC-labelled probe for the reference gene assay and one FAM-labelled for the ssODN sequence assay). These reaction mixes were loaded either into DG8 cartridges together with 70 μl droplet oil per sample and the droplets generated using the QX100 Droplet Generator or loaded in plate format into the Bio-Rad QX200 AutoDG and the droplets generated as per the manufacturer’s instructions. Post droplet generation, the oil/reagent emulsion was transferred to a 96-well semi-skirted plate (Eppendorf), and the samples were amplified on a Bio-Rad C1000 Touch thermocycler (95 °C for 10 min, followed by 40 cycles of 94 °C for 30 s and 58 °C for 60 s, with a final elongation step of 98 °C for 10 min, where all temperature ramping was set to 2.5 °C/s). The plate containing the droplet amplicons was subsequently loaded into the QX200 Droplet Reader (Bio-Rad). Standard reagents and consumables supplied by Bio-Rad were used, including cartridges and gaskets, droplet generation oil and droplet reader oil. Copy numbers were assessed using the QuantaSoft software using at least 10,000 accepted droplets per sample. The copy numbers were calculated by applying Poisson statistics to the fraction of end-point positive reactions, and the 95% confidence interval of this measurement is shown.

## Additional files

Additional file 1: **Table S1.** Sequences of reagents used in the study. The table shows the sequences of the oligonucleotides and lssDNA donors, primers and TaqMan assays employed in this study. LoxP sites (for all conditional projects) and point mutations (for Gckr and Rims1 project) are underlined. Sequences added for diagnostic (for all conditional projects except Syt7) and silent mutations (for Gckr and Rims1 project) are shown in italics. For the plasmids, sequences flanked by and including homology arms are shown. The ddPCR reference copy counting assay is labelled with VIC. All other ddPCR copy counting assays are labelled with fluorescein amidite (FAM). Copy counting assays labelled as UNIV ddPCR assays recognize both WT and engineered alleles; MUT ddPCR assays recognize engineered allele only. **Table S2.** Production of founders for conditional alleles. The table shows the numbers of embryos and animals involved in mutagenesis attempts employing the injection of CRISPR/Cas9 reagents and lssDNA donors. **Table S3.** Generation of conditional alleles employing different donor types. The table shows the numbers of embryos and animals involved in mutagenesis attempts employing the injection of CRISPR/Cas9 reagents and oligonucleotides, plasmids or lssDNA donors. The results of the analysis of the founders obtained from these attempts are also summarized. **Table S4.** Generation of a Rims1^R655H^ point mutation. Further genotype screening data for this project are shown in Additional file 18: Figure S16 and Additional file 19: Figure S17. **Table S5.** Analysis of the Rims1^R655H^ project. The table details the results of screening of five positive F_0_ animals obtained for the generation of a Rims1^R655H^ point mutation and the subsequent characterization of the F_1_ animals obtained from mating of these F_0_ animals to WT mice. **Table S6.** Nomenclature of new mouse lines established in the course of the study. (XLS 81 kb)

Additional file 2: **Figure S1.** Screening by Sanger sequencing of animals for the generation of a *Syt7* conditional allele. The figure shows the sequencing traces from PCR products amplified from founder Syt7-4 (a) and founder Syt7-8 (b) that reveal the integration of two loxP sites in both animals. Note that Syt7-8 appears to be homozygous (a single trace detected), while Syt7-4 appears to contain at least two different alleles. The PCR products from which the sequence traces were derived are shown in Fig. 1. (PNG 377 kb)

Additional file 3: **Figure S2.** Additional animal analysis information. (DOCX 19408 kb)

Additional file 4: **Figure S3.** The figure shows the designs of reagents employed for the generation of conditional alleles. Red triangles mark loxP sites. RNA is transcribed in vitro from a double-stranded DNA template containing the T7 promoter and the donor sequence. The resulting RNA is reverse-transcribed employing a primer that is specific to the donor sequence. Additional sequences (orange boxes, marked as universal) were added to the design for the purpose of facilitating initial screening of animals employing restriction enzyme sites and/or validated primer pairs, with the exception of the *Syt7* conditional allele (described in Fig. 1). (PNG 91 kb)

Additional file 5: **Figure S4.** Analysis of the Ikzf2 project. PCR amplification of the genomic region of interest from (a, b) F_0_ animals and (f, g) Ikzf2-2’s offspring with (a, f) Ikzf2-F3 and Ikzf2-3R2 primers (1594-bp amplicon) and (b, g) LoxPF and LoxPR primers (906-bp amplicon) from biopsies. (a, b, f, g) Animals’ IDs are shown. + is positive control amplified from an unrelated (a) WT, (b) plasmid template. Sequencing of PCR amplicon from (c) the founder Ikzf2-2, (h) Ikzf2-2.1f and (i) Ikzf2-2.1 h with Ikzf2-F3 and Ikzf2-3R2 primers. LoxP sequences are highlighted in blue. (d) ID and outcome of PCR analysis of the region of interest and the conclusion for each F_0_ individual. (e) ID, outcome of sequencing and copy counting of the region of interest as well as the conclusion for each individual of the first litter obtained by mating Ikzf2-2 with a WT mouse. *Animal mated; **deletion not picked up by Ikzf2 PCR, likely encompassing at least one primer sequence; ***allele detailed in Additional file 14: Figure S13. Evidence of deletion is highlighted in blue. L1 = 1 kb DNA molecular weight ladder (thick band is 3 kb). Sequencing data showing a correct conditional allele are shown in Additional file 3: Figure S2d. Sequencing data showing the presence of a deletion allele in founders Ikzf2-4 and Ikzf2-8 are shown in Additional file 3: Figure S2b and c. (PNG 1031 kb)

Additional file 6: **Figure S5.** Analysis of the Syt4 project. PCR amplification of the genomic region of interest with (a) Syt4-F2 and Syt4-R1 primers (2088-bp amplicon) and (b) Syt4-LoxPF and Syt4-LoxPR primers (1395-bp amplicon) from F_0_ animal biopsies. (c) Sequencing of PCR amplicon obtained from founder Syt4-29 with Syt4-F2 and Syt4-R1. LoxP sequences are highlighted in *blue*. (d) ID, PCR analysis of the region of interest and conclusion for each F_0_ individual are shown. *Syt4-29 was mated for cKO allele transmission. **Syt4-37 was identified as having a random insertion of the donor, as sequencing of the Syt4 PCR amplicon obtained from Syt4-37 shows no loxP, suggesting a random integration of the donor, Additional file 3: Figure S2j. (e) Details of the first litter obtained by mating Syt4-29 with a WT mouse. ID, outcome of sequencing and copy counting of the region of interest and the conclusion for each individual are shown. PCR amplification of region of interest with Syt4-F2 and Syt4-R1 primers (2088-bp amplicon (f) and LoxPF and LoxPR primers (1395-bp amplicon (g) from biopsies taken from founder Syt4-29’s offspring. (h) Sequencing data obtained from Syt4-29.1a. (a, b, f, g) Animal IDs are shown. + is positive control amplified from an unrelated WT (a, f). L1 = 1 kb DNA molecular weight ladder (thick band is 3 kb). L2 = 100 bp DNA molecular weight ladder (thick bands are 1000 and 500 bp). Sequencing data showing a correct conditional allele are shown in Additional file 3: Figure S2k. Sequencing data showing the transmission of a deletion allele by founder Syt4-17 are shown in Additional file 3: Figure S2e, f and g. Sequencing data illustrating the possible insertion of loxP in Syt-28 and the transmission of an illegitimate repair are shown in Additional file 3: Figure S2i and j. (PNG 1045 kb)

Additional file 7: **Figure S6.** Analysis of the Usp45 project. The figure shows the PCR amplification of the genomic region of interest with (a) Usp45-F1 and Usp45-R3 primers (1440-bp amplicon) and (b) LoxPF and LoxPR primers (741-bp amplicon) from biopsies taken from the F_0_ animals. (c) The panels show the Usp45 PCR amplicon generated from the Usp45-18 can be sequenced with LoxPF and LoxPR primers, demonstrating the presence of loxP on locus. (d) The table details the F_0_ animals obtained. The ID and outcome of PCR analysis of the region of interest as well as the conclusion for each individual are shown. Usp45-18 was mated for cKO allele transmission. (e) The table details three litters obtained by mating Usp45-18 with a WT mouse. The ID, outcome of sequencing the region of interest and the conclusion for each individual are shown. PCR amplification of region of interest with Usp45-F1 and Usp45-R3 primers (1440-bp amplicon (f) and LoxPF and LoxPR primers (741-bp amplicon (g) from biopsies taken from Usp45-18’s offspring. Animal IDs are shown. + is positive control amplified from an unrelated WT (a, f). L1 = 1 kb DNA molecular weight ladder (thick band is 3 kb). Sequencing data obtained from Usp45-18.1a and Usp45-18.1b are shown in Additional file 3: Figure S2l and m. (a) Litter 3 died prior to biopsy age. (b) Deletion affecting the region recognized by the TaqMan assay. (c) Litter died prior to biopsy age. (d) Copy number counting of mutated sequence. n.d. = not determined. Further data are displayed in Additional file 3: Figure S2. (PNG 618 kb)

Additional file 8: **Figure S7.** Analysis of the Rapgef5 project. PCR amplification of the genomic region of interest with (a) Rapgef5-F1 and Rapgef5-R1 primers (1365-bp amplicon) and (b) LoxPF and LoxPR primers (724-bp amplicon) from biopsies taken from the F_0_ animals. (a, b) Animal IDs are shown. + is positive control amplified from an unrelated (a) WT, (b) conditional floxed animal. L1 = 1 kb DNA molecular weight ladder (thick band is 3 kb). (c) Panel shows the sequencing of PCR amplicon obtained from the Rapgef5-14 with Rapgef5-F1 and Rapgef5-R1 primers. LoxP sequences are highlighted in *blue*. (d) The table details the F_0_ animals obtained. The ID and outcome of PCR analysis of the region of interest and the conclusion for each individual are shown. Founder Rapgef5-14 died without offspring. Sequencing data showing the deletion allele identified in Rapgef5-3 are shown in Additional file 3: Figure S2n. (PNG 587 kb)

Additional file 9: **Figure S8.** Analysis of the Cx3cl1 project. PCR amplification of the genomic region of interest with (a) Cx3cl1-F1 and Cx3cl1-R1 primers (1483-bp amplicon) and (b) LoxPF and LoxPR primers (835-bp amplicon) from biopsies taken from the F_0_ animals. (c) The panels show the sequencing of PCR amplicon obtained from animal Cx3cl1-10 with Cx3cl1-F1 and Cx3cl1-R1. LoxP sequences are highlighted in *blue*. (d) The table details the F_0_ animals obtained. The ID and outcome of PCR analysis of the region of interest, as well as the conclusion for each individual are shown. Three founders are mated for cKO allele transmission (LoxP PCR positive and sequence of complex mosaic). PCR amplification of region of interest with (e) Cx3cl1-F1 and Cx3cl1-R1 primers (1483-bp amplicon) and LoxPF and LoxPR primers (835-bp amplicon) from biopsies taken from Cx3cl1-10’s offspring. (f) The table details the first litter obtained by mating Cx3cl1-10 with a WT mouse. The ID, outcome of sequencing the region of interest, copy counting of the region of interest and the conclusion for each individual are shown. (g) The panel shows an alignment of the sequencing data obtained from Cx3cl1-10.1a. Blue 5′homology arm; orange universal sequences for diagnostics; green critical region with exon in capitals; red loxP sites; grey 3′homology arm. (a, b, e) Animal IDs are shown. + is positive control amplified from an unrelated (a) WT, (b) conditional floxed animal. L1 = 1 kb DNA molecular weight ladder (thick band is 3 kb). Sequencing data showing examples of illegitimately repaired conditional alleles are shown in Additional file 3: Figure S2o and p. (PNG 1075 kb)

Additional file 10: **Figure S9.** Analysis of the 6430573F11Rik project. PCR amplification of genomic DNA of (a) F_0_ animals, (f) 6430573F11Rik-11’s offspring or (i) 6430573F11Rik-28’s offspring with (a, f) 6430573F11Rik-F3 and 6430573F11Rik-R2 (1721-bp amplicon) and (b, f) LoxPF and LoxPR (999-bp amplicon). Sequencing of PCR amplicons from (c) 6430573F11Rik-11 and (g) 6430573F11Rik-11.1a with 6430573F11Rik-F3 and 6430573F11Rik-R2. LoxPs are in blue. ID, outcome of PCR analysis and conclusion for (d) each F_0_ animal and (e) the first litter obtained by mating 6430573F11Rik-11 with a WT mouse. Two founders were mated for cKO GLT. *Mated; ⁑no evidence of loxP in 6430573F11Rik amplicon, suggesting donor integrated randomly (6430573F11Rik-28 sequence trace in Additional file 3: Figure S2q). (g) Only WT sequence is found, indicating random donor insertion. (f, i) Animal IDs are shown. + is positive control from unrelated WT and conditional floxed animal for 6430573F11Rik and LoxP PCR, respectively. L1 = 1 kb DNA molecular weight ladder (thick band is 3 kb). (h) First litter obtained by mating 6430573F11Rik-28 with a WT mouse. ID, outcome of sequencing and copy counting of the region of interest and the conclusion for each individual. (j) Sequencing of amplicons obtained with 6430573F11Rik-F3 and 6430573F11Rik-R2 and 6430573F11Rik-28.1a. Only WT sequence is found, indicating random donor insertion. Sequencing of deletion allele in founder 6430573F11Rik-6, summary of analysis of F_1_ animals derived from 6430573F11Rik-6 and transmitted deletion allele are shown in Additional file 3: Figure S2r, s and t. (PNG 1011 kb)

Additional file 11: **Figure S10.** Analysis of the Acvr2b project. The figure shows the PCR amplification of the genomic region of interest with (a) Acvr2b-F1 and Acvr2b-R1 primers (2178 bp) and (b) LoxPF and LoxPR primers (1689 bp) from biopsies taken from the F_0_ animals. (a, b) Animal IDs are shown. + is positive control amplified from an unrelated (a) WT, (b) conditional floxed animal. L1 = 1 kb DNA molecular weight ladder (thick band is 3 kb). (c) The table details the F_0_ animals obtained. The ID and outcome of PCR analysis of the region of interest as well as the conclusion for each individual are shown. (PNG 255 kb)

Additional file 12: **Figure S11.** Analysis of the Inpp5k project. The figure shows the PCR amplification of the genomic region of interest with (a) Inpp5k-F1 and Inpp5k-R1 primers (1705-bp amplicon) and (b) LoxPF and LoxPR primers (1194-bp amplicon) from biopsies taken from the F_0_ animals. Animal IDs are shown. + is positive control amplified from an unrelated WT and conditional floxed animal for the Inpp5k and LoxP PCR, respectively. L1 = 1 kb DNA molecular weight ladder (thick band is 3 kb). (c) Sequencing chromatogram of PCR amplicons obtained from Inpp5k-7 with Inpp5k-F1 and Inpp5k-R1. LoxP sequence is highlighted in blue. (d) The table details the F_0_ animals obtained. The ID, outcome of PCR analysis of the region of interest and the conclusion for each individual are shown. Two founders are mated for cKO allele transmission (LoxP PCR positive and sequence of complex mosaic). *Mated as loxP presence confirmed by sequencing of Inpp5k PCR amplicon. (e) First litter obtained by mating Inpp5k-7 and Inpp5k-8 with a WT mouse. The ID, outcome of sequencing the region of interest and the conclusion for each individual are shown. PCR amplification of region of interest with (f) Inpp5k-F1 and Inpp5k-R1 primers (1705-bp amplicon) and (g) LoxPF and LoxPR primers (1194-bp amplicon) from biopsies taken from Inpp5k-7’s and Inpp5k-8’s offspring. Animal IDs are shown. + is positive control amplified from an unrelated WT and conditional floxed animal for the Inpp5k and LoxP PCR, respectively. L1 = 1 kb DNA molecular weight ladder (thick band is 3 kb). Panels illustrate the sequencing data from amplicons obtained from Inpp5k-7.1b (h, i, j) and Inpp5k-8.1c (k, l) genomic DNA with (h, i, k) Inpp5k-F1 and (j, l) Inpp5k-R1 primers. (PNG 877 kb)

Additional file 13: **Figure S12.** Examples of unexpected point mutations in the F_0_ animals obtained from the co-injection of CRISPR/Cas9 reagents and lssDNA in 6430573F11Rik (a) and Cx3cl1 (b and c) projects. Blue 5′ homology arm; orange universal sequences for diagnostics; green critical region with exon in capitals; red loxP sites; grey 3′ homology arm. Unexpected point mutations are detected by Sanger sequencing of amplicons generated with primers external to the donor; (a) shows one intronic SNP in floxed critical region, (b) shows two intronic nucleotide changes (black arrows, grey highlight) and one coding nucleotide change (red arrow, pink highlight) which was found associated with (c) SNP in 3’ loxP site. Mutations are highlighted on the sequence alignment (a) and seen on the sequence chromatograms (b and c). (PNG 1332 kb)

Additional file 14: **Figure S13.** Unexpected outcome of CRISPR/Cas9-aided mutagenesis. The figure illustrates an example of a rearranged allele obtained from the co-injection of CRISPR/Cas9 reagents and lssDNA to generate a conditional *Ikzf2* allele. Panel (a) shows the design of the lssDNA donor compared to the WT sequence. HA homology arm, BP breakpoint (genomic sequence removed in the intended floxed allele). Panel (b) shows sequencing of an F_1_ (Ikzf2–2.1e) that bears a recombined allele where the critical region and a loxP site are lost (allele with major representation) and a WT allele (with minor representation). (PNG 309 kb)

Additional file 15: **Figure S14.** Design of a Gckr^P446L^ point mutation. Figure illustrates the changes designed at the nucleotide and proteomic levels with the mutagenesis strategy employing (a) oligonucleotides and (b) lssDNA. Coding sequences are highlighted in pink, engineered P446L change is highlighted in black with yellow text, silent mutations are highlighted in grey and sgRNA sequences are highlighted in green. Primers external to the donors employed for mutant analysis are also shown in blue and detailed in Additional file 1: Table S1. (PNG 1857 kb)

Additional file 16: The file contains the raw sequencing data obtained from the founders generated for the Gckr^P446L^ point mutation. (ZIP 14449 kb)

Additional file 17: **Figure S15.** Design of a Rims1^R655H^ point mutation. The figure illustrates the changes designed at the nucleotide and proteomic levels with the mutagenesis strategy employing (a) oligonucleotides and (b) lssDNA. Coding sequences are translated into protein sequences above annotated exon. Note that the region containing Rims1 is not entirely accurate in the GRCm38 assembly. We have re-sequenced this region prior to designing of the mutant (primers shown in Additional file 1: Table S1). (PNG 521 kb)

Additional file 18: **Figure S16.** Generation of a point mutation in *Rims1* with ssODN donors. (a) The table details the F_0_ animals obtained for generation of Rims1 mutant with ssODN donors. The ID and outcome of sequencing the region of interest, as well as the conclusion for each individual are shown. (b) PCR amplification of region of interest with Rims1-F1 and Rims1-R1 primers (241 bp) from biopsies taken from the F_0_ animals. Sequences of Rims1-ODN-151 mosaic and of sub-cloned amplicons are shown in Additional file 3: Figure S2u and v, demonstrating the presence of the desired mutation in this animal that was therefore mated. (c) PCR amplification of region of interest with Rims1-F1 and Rims1-R1 primers (241 bp) from biopsies taken from Rims1-ODN-151’s offspring. Animal IDs are shown. + is positive control amplified from an unrelated WT animal. L1 = 1 kb DNA molecular weight (thick bands are 3 kb); L2 = 100 bp DNA molecular weight ladder (thick bands are 1000 and 500 bp). (d) The table details the first litter obtained by mating Rims1-ODN-151 with a WT mouse. The ID, outcome of sequencing the region of interest and copy counting of the region of interest as well as the conclusion for each individual are shown. Sequencing of Rims1-ODN-151.1g is shown in Additional file 3: Figure S2w and illustrates the failure of transmission of the desired allele. (PNG 893 kb)

Additional file 19: **Figure S17.** Generation of a point mutation in *Rims1* with a lssDNA donors. (a) PCR amplification of region of interest with Rims1-F2 and Rims1-R2 primers (647 bp) from biopsies taken from the F_0_ animals. Animal IDs are shown. + is positive control amplified from an unrelated WT animal. L1 = 1 kb DNA molecular weight ladder (thick band is 3 kb). (b) Sequencing of amplicon obtained from the Rims1-lss-2, Rims1-lss-20, Rims1-lss-21 and Rims1-lss-36 animals: point mutation is observed (*blue highlight*) when sequencing the Rims1-F2 primer. (c) The table details the F_0_ animals obtained for generation of Rims1 mutant with lssDNA donors. The ID, outcome of sequencing the region of interest and the conclusion for each individual are shown. (d) The table details the first litter obtained by mating Rims1-lss-36 with a WT mouse. The ID, outcome of sequencing the region of interest, copy counting of the region of interest and conclusion for each individual are shown. (e) PCR amplification of region of interest with Rims1-F3 and Rims1-R3 primers (647 bp) from biopsies taken from Rims-lss-36’s offspring. Animal IDs are shown. + is a positive control amplified from an unrelated WT animal. L2 = 100 bp DNA molecular weight ladder (thick bands are 1000 and 500 bp). (f) Sequencing of amplicon obtained from Rims1-lss-36.1a, legitimate repair observed (*blue highlight*) when sequencing both directions (Rims1-F3 and Rims1-R3 primers). (g) Alignment of Rims1-lss-36-1a offspring, legitimate repair aligned against WT allele. R655H coding change highlighted in red. Grey background with red text highlights silent mutations introduced by long donor. (PNG 1287 kb)

Additional file 20: The file contains the raw sequencing data obtained from the founders generated for the Rims1^R655H^ point mutation. (ZIP 21747 kb)

## Abbreviations

bp: Base pair
Cas9: CRISPR-associated protein 9
cKO: Conditional knock-out
CRISPR: Clustered regularly interspaced short palindromic repeats
ddPCR: Droplet digital PCR
GLT: Germline transmission
HA: Homology arm
HR: Homologous recombination
IVT: In vitro transcription
KI: Knock-in
KO: Knock-out
lssDNA: Long single-stranded DNA
NHEJ: Non-homologous end joining
nt: Nucleotide
PAM: Protospacer adjacent motif
QC: Quality control
QTL: Quantitative trait locus
RGEN: RNA-guided engineered nuclease
sgRNA: Single guide RNA
ssODN: Single-stranded oligodeoxynucleotide
WGS: Whole genome sequencing
WT: Wild-type
