# Supplementary figures and images for: Application of long single-stranded DNA donors in genome editing: generation and validation of mouse mutants
Source: BMC Biol. 2018 Jun 21;16:70. doi: 10.1186/s12915-018-0530-7 (PMC6011369; doi:10.1186/s12915-018-0530-7)

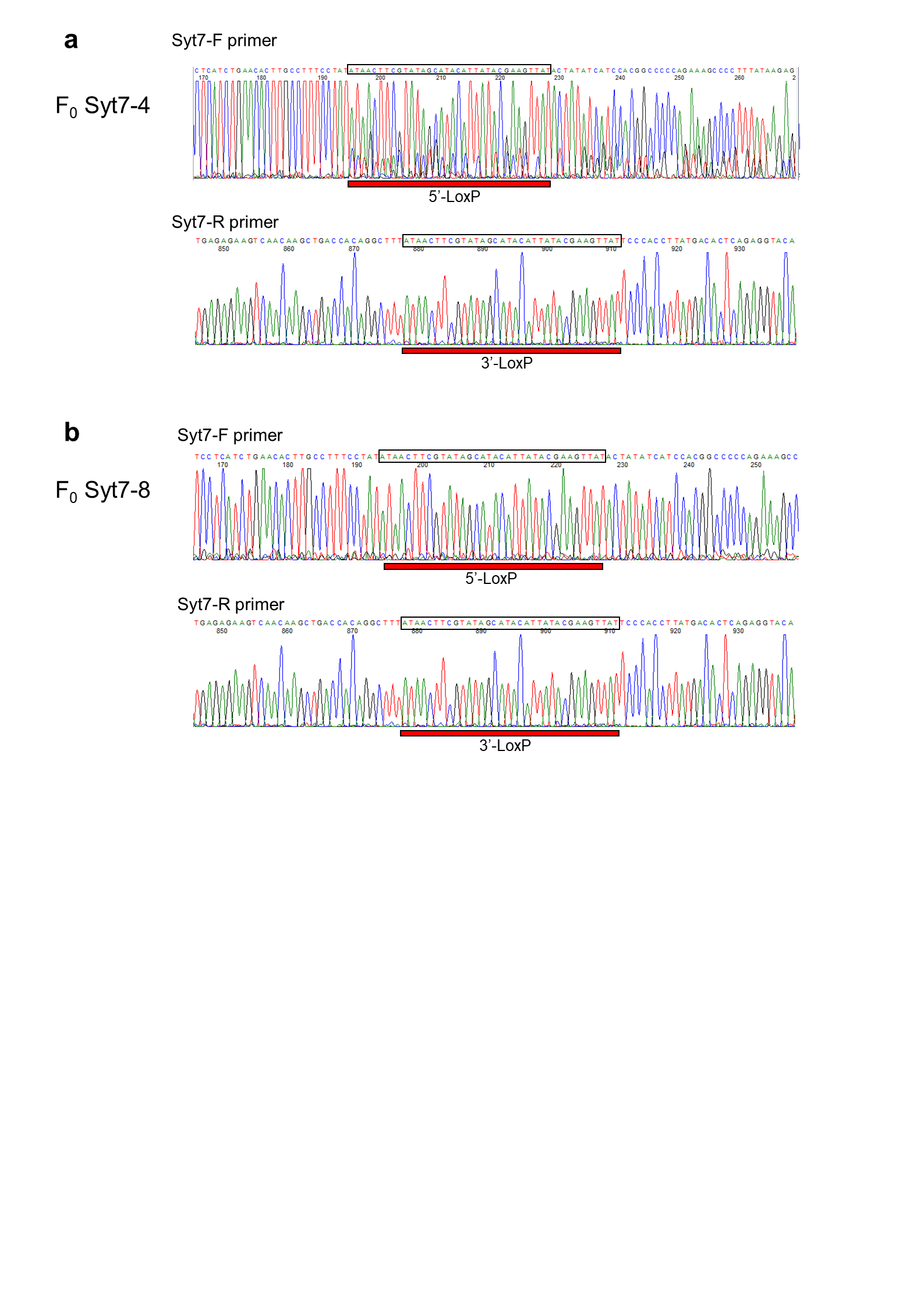

Supplement: Supplementary file 2 — Figure S1. Screening by Sanger sequencing of animals for the generation of a Syt7 conditional allele. The figure shows the sequencing traces from PCR products amplified from founder Syt7-4 (a) and founder Syt7-8 (b) that reveal the integration of two loxP sites in both animals. Note that Syt7-8 appears to be homozygous (a single trace detected), while Syt7-4 appears to contain at least two different alleles. The PCR products from which the sequence traces were derived are shown in Fig. 1. (PNG 377 kb) [file 12915_2018_530_MOESM2_ESM.png]

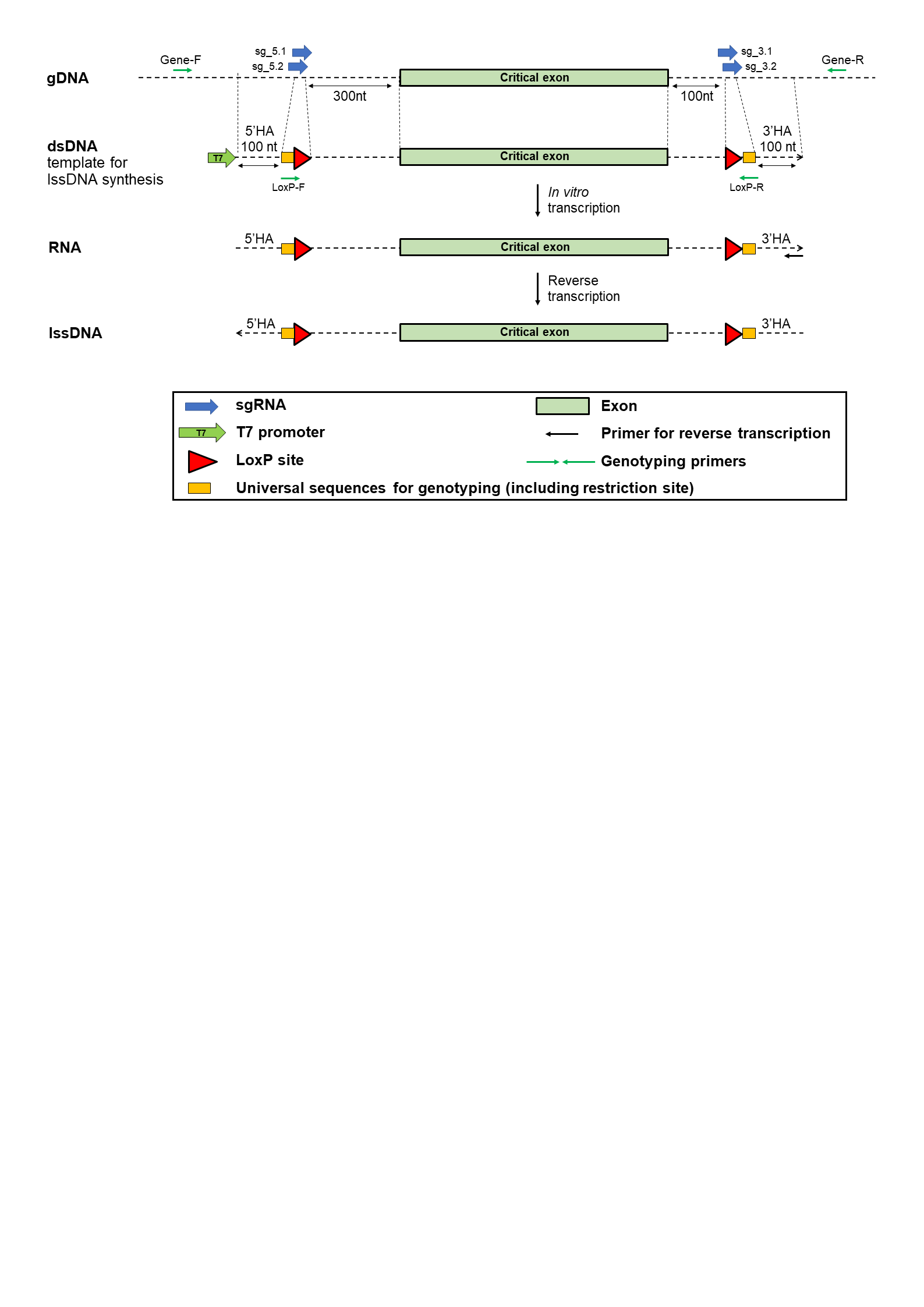

Supplement: Supplementary file 4 — Figure S3. The figure shows the designs of reagents employed for the generation of conditional alleles. Red triangles mark loxP sites. RNA is transcribed in vitro from a double-stranded DNA template containing the T7 promoter and the donor sequence. The resulting RNA is reverse-transcribed employing a primer that is specific to the donor sequence. Additional sequences (orange boxes, marked as universal) were added to the design for the purpose of facilitating initial screening of animals employing restriction enzyme sites and/or validated primer pairs, with the exception of the Syt7 conditional allele (described in Fig. 1). (PNG 91 kb) [file 12915_2018_530_MOESM4_ESM.png]

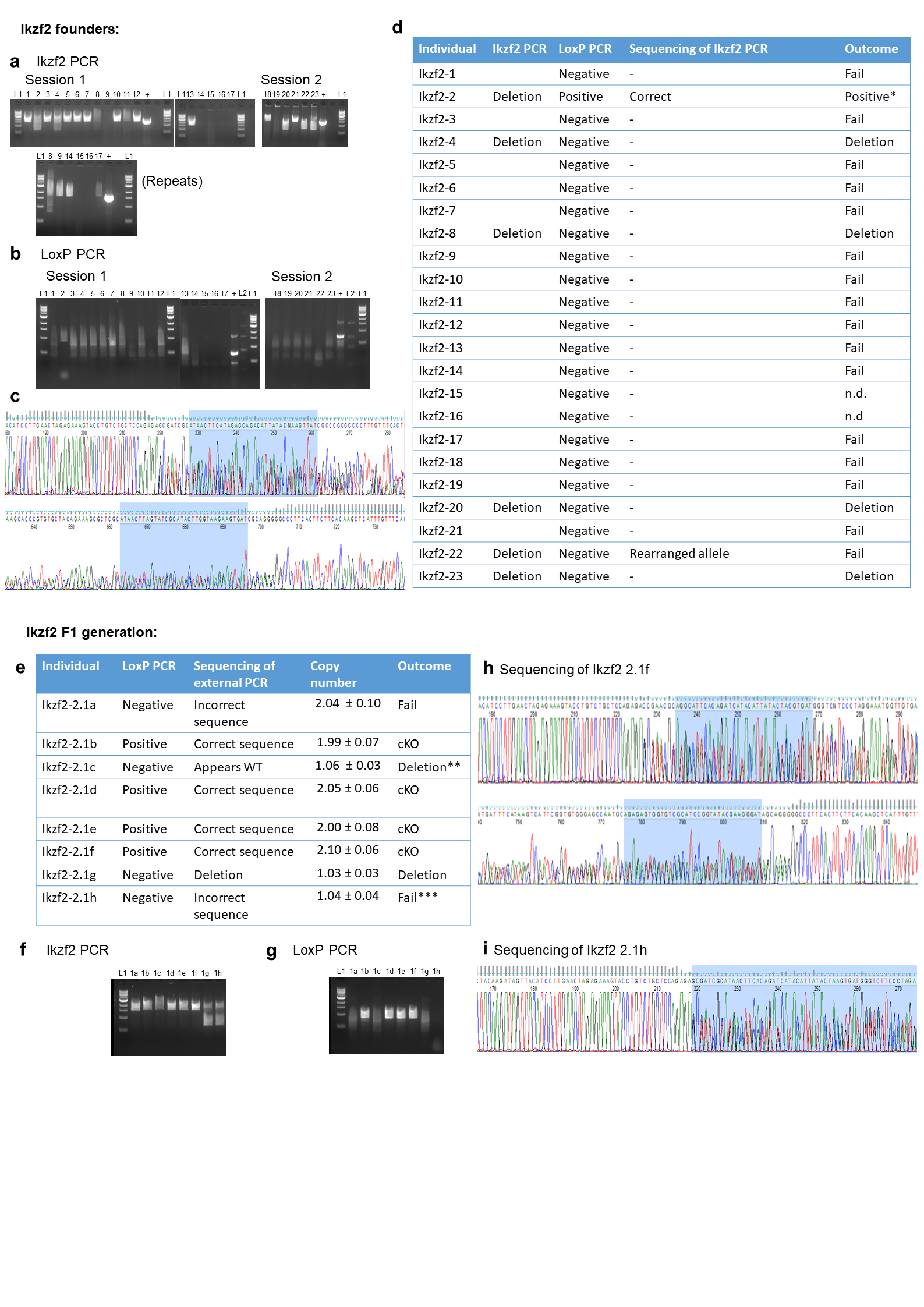

Supplement: Supplementary file 5 — Figure S4. Analysis of the Ikzf2 project. PCR amplification of the genomic region of interest from (a, b) F0 animals and (f, g) Ikzf2-2’s offspring with (a, f) Ikzf2-F3 and Ikzf2-3R2 primers (1594-bp amplicon) and (b, g) LoxPF and LoxPR primers (906-bp amplicon) from biopsies. (a, b, f, g) Animals’ IDs are shown. + is positive control amplified from an unrelated (a) WT, (b) plasmid template. Sequencing of PCR amplicon from (c) the founder Ikzf2-2, (h) Ikzf2-2.1f and (i) Ikzf2-2.1 h with Ikzf2-F3 and Ikzf2-3R2 primers. LoxP sequences are highlighted in blue. (d) ID and outcome of PCR analysis of the region of interest and the conclusion for each F0 individual. (e) ID, outcome of sequencing and copy counting of the region of interest as well as the conclusion for each individual of the first litter obtained by mating Ikzf2-2 with a WT mouse. *Animal mated; **deletion not picked up by Ikzf2 PCR, likely encompassing at least one primer sequence; ***allele detailed in Additional file 14: Figure S13. Evidence of deletion is highlighted in blue. L1 = 1 kb DNA molecular weight ladder (thick band is 3 kb). Sequencing data showing a correct conditional allele are shown in Additional file 3: Figure S2d. Sequencing data showing the presence of a deletion allele in founders Ikzf2-4 and Ikzf2-8 are shown in Additional file 3: Figure S2b and c. (PNG 1031 kb) [file 12915_2018_530_MOESM5_ESM.png]

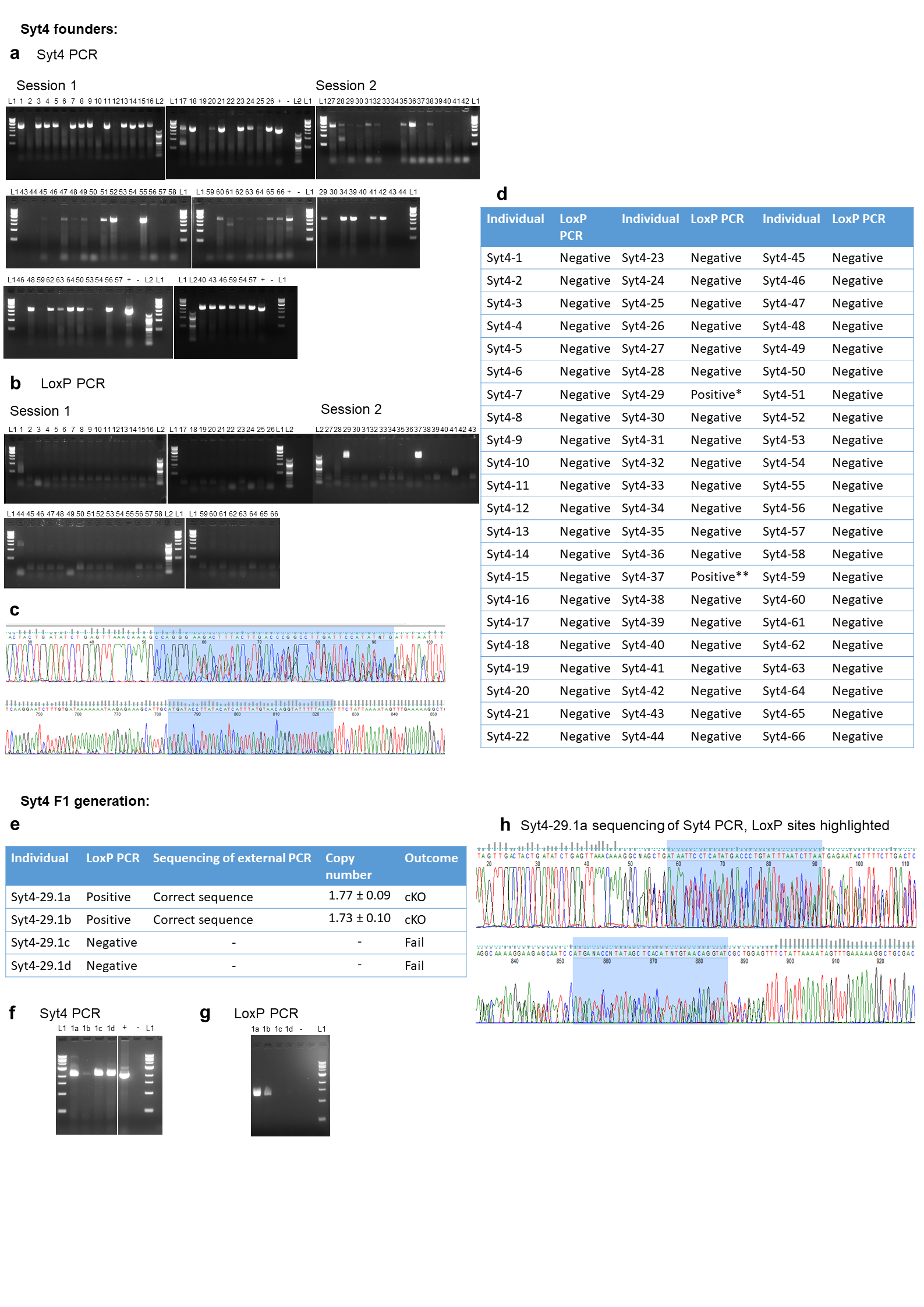

Supplement: Supplementary file 6 — Figure S5. Analysis of the Syt4 project. PCR amplification of the genomic region of interest with (a) Syt4-F2 and Syt4-R1 primers (2088-bp amplicon) and (b) Syt4-LoxPF and Syt4-LoxPR primers (1395-bp amplicon) from F0 animal biopsies. (c) Sequencing of PCR amplicon obtained from founder Syt4-29 with Syt4-F2 and Syt4-R1. LoxP sequences are highlighted in blue. (d) ID, PCR analysis of the region of interest and conclusion for each F0 individual are shown. *Syt4-29 was mated for cKO allele transmission. **Syt4-37 was identified as having a random insertion of the donor, as sequencing of the Syt4 PCR amplicon obtained from Syt4-37 shows no loxP, suggesting a random integration of the donor, Additional file 3: Figure S2j. (e) Details of the first litter obtained by mating Syt4-29 with a WT mouse. ID, outcome of sequencing and copy counting of the region of interest and the conclusion for each individual are shown. PCR amplification of region of interest with Syt4-F2 and Syt4-R1 primers (2088-bp amplicon (f) and LoxPF and LoxPR primers (1395-bp amplicon (g) from biopsies taken from founder Syt4-29’s offspring. (h) Sequencing data obtained from Syt4-29.1a. (a, b, f, g) Animal IDs are shown. + is positive control amplified from an unrelated WT (a, f). L1 = 1 kb DNA molecular weight ladder (thick band is 3 kb). L2 = 100 bp DNA molecular weight ladder (thick bands are 1000 and 500 bp). Sequencing data showing a correct conditional allele are shown in Additional file 3: Figure S2k. Sequencing data showing the transmission of a deletion allele by founder Syt4-17 are shown in Additional file 3: Figure S2e, f and g. Sequencing data illustrating the possible insertion of loxP in Syt-28 and the transmission of an illegitimate repair are shown in Additional file 3: Figure S2i and j. (PNG 1045 kb) [file 12915_2018_530_MOESM6_ESM.png]

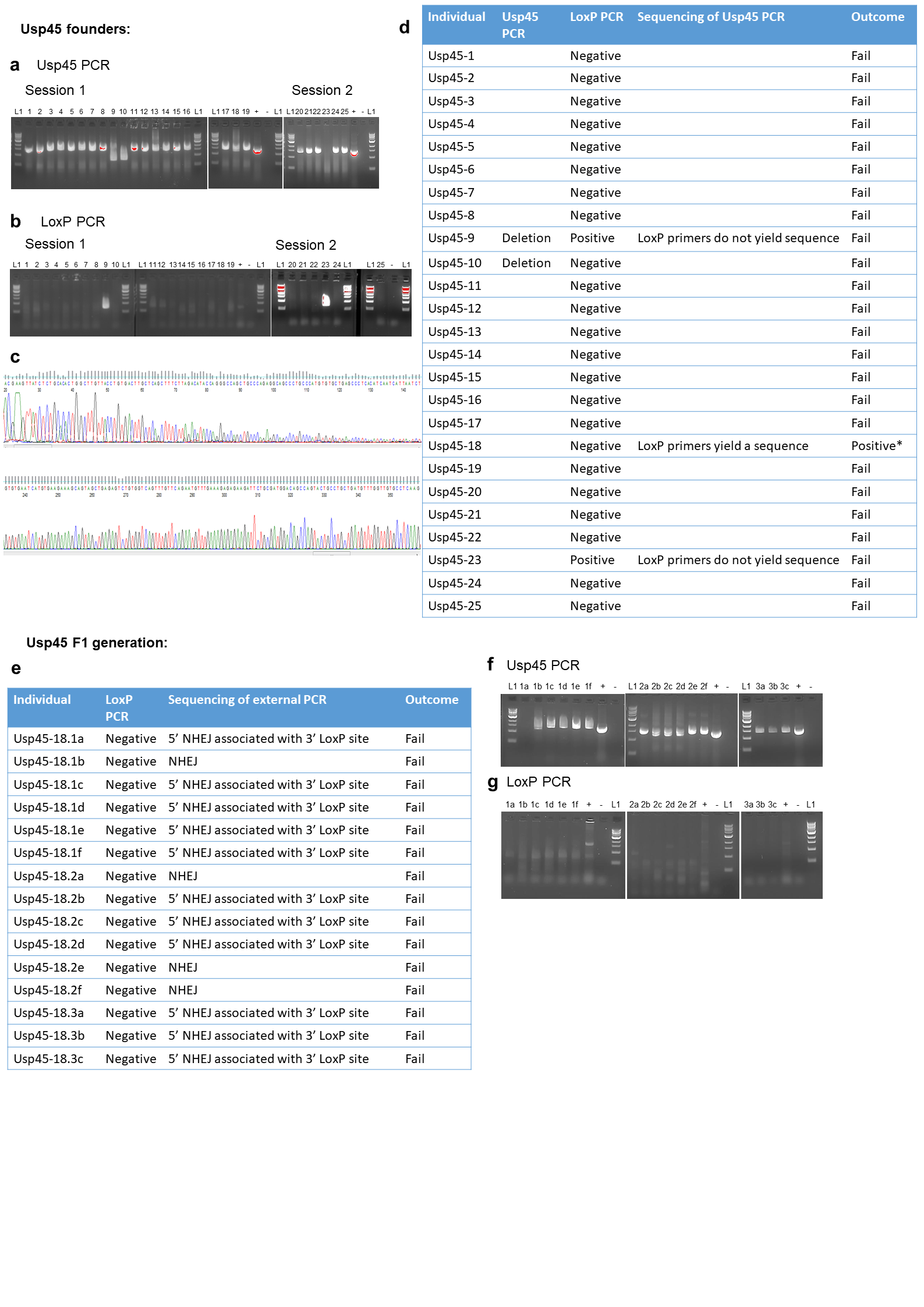

Supplement: Supplementary file 7 — Figure S6. Analysis of the Usp45 project. The figure shows the PCR amplification of the genomic region of interest with (a) Usp45-F1 and Usp45-R3 primers (1440-bp amplicon) and (b) LoxPF and LoxPR primers (741-bp amplicon) from biopsies taken from the F0 animals. (c) The panels show the Usp45 PCR amplicon generated from the Usp45-18 can be sequenced with LoxPF and LoxPR primers, demonstrating the presence of loxP on locus. (d) The table details the F0 animals obtained. The ID and outcome of PCR analysis of the region of interest as well as the conclusion for each individual are shown. Usp45-18 was mated for cKO allele transmission. (e) The table details three litters obtained by mating Usp45-18 with a WT mouse. The ID, outcome of sequencing the region of interest and the conclusion for each individual are shown. PCR amplification of region of interest with Usp45-F1 and Usp45-R3 primers (1440-bp amplicon (f) and LoxPF and LoxPR primers (741-bp amplicon (g) from biopsies taken from Usp45-18’s offspring. Animal IDs are shown. + is positive control amplified from an unrelated WT (a, f). L1 = 1 kb DNA molecular weight ladder (thick band is 3 kb). Sequencing data obtained from Usp45-18.1a and Usp45-18.1b are shown in Additional file 3: Figure S2l and m. (a) Litter 3 died prior to biopsy age. (b) Deletion affecting the region recognized by the TaqMan assay. (c) Litter died prior to biopsy age. (d) Copy number counting of mutated sequence. n.d. = not determined. Further data are displayed in Additional file 3: Figure S2. (PNG 618 kb) [file 12915_2018_530_MOESM7_ESM.png]

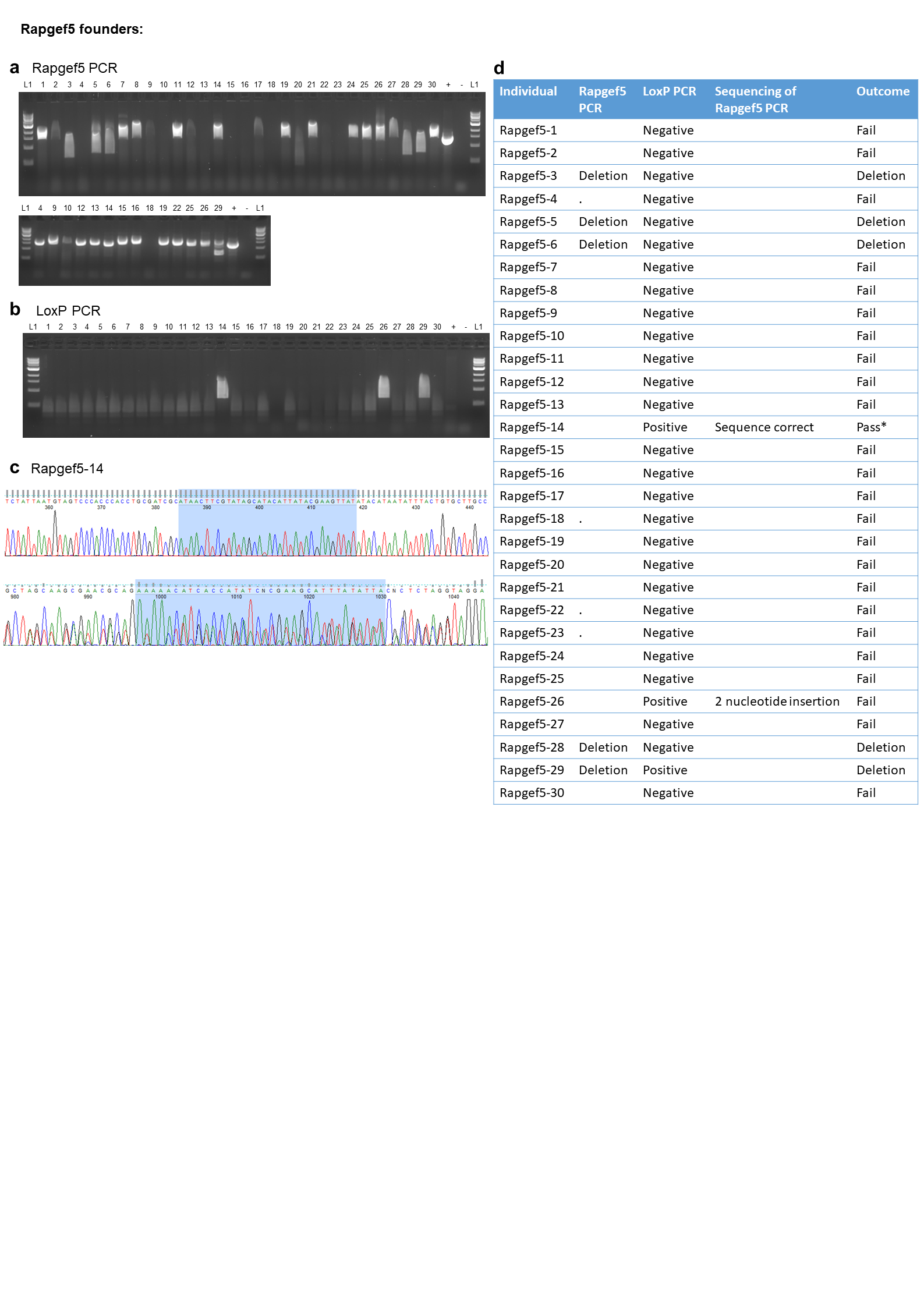

Supplement: Supplementary file 8 — Figure S7. Analysis of the Rapgef5 project. PCR amplification of the genomic region of interest with (a) Rapgef5-F1 and Rapgef5-R1 primers (1365-bp amplicon) and (b) LoxPF and LoxPR primers (724-bp amplicon) from biopsies taken from the F0 animals. (a, b) Animal IDs are shown. + is positive control amplified from an unrelated (a) WT, (b) conditional floxed animal. L1 = 1 kb DNA molecular weight ladder (thick band is 3 kb). (c) Panel shows the sequencing of PCR amplicon obtained from the Rapgef5-14 with Rapgef5-F1 and Rapgef5-R1 primers. LoxP sequences are highlighted in blue. (d) The table details the F0 animals obtained. The ID and outcome of PCR analysis of the region of interest and the conclusion for each individual are shown. Founder Rapgef5-14 died without offspring. Sequencing data showing the deletion allele identified in Rapgef5-3 are shown in Additional file 3: Figure S2n. (PNG 587 kb) [file 12915_2018_530_MOESM8_ESM.png]

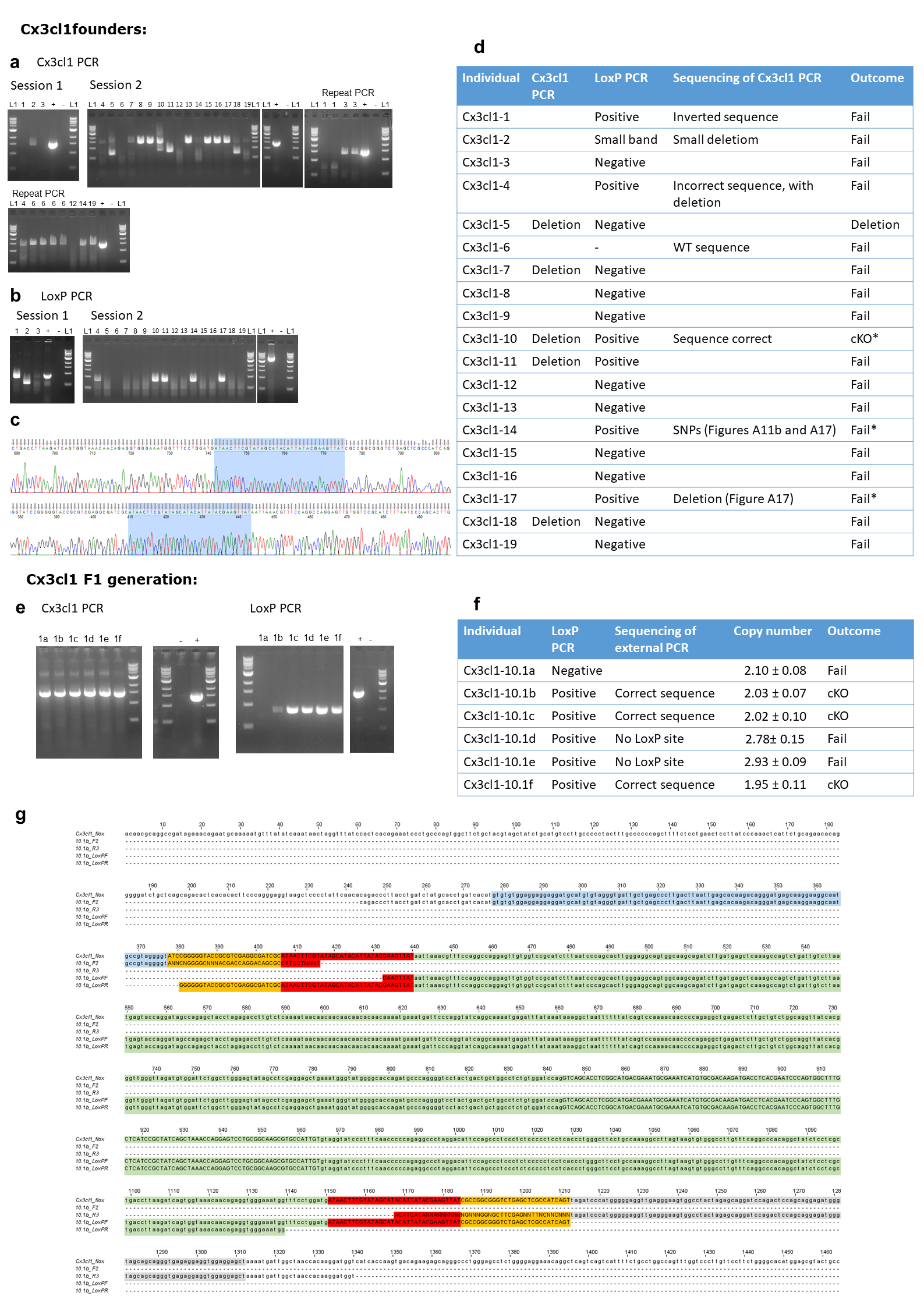

Supplement: Supplementary file 9 — Figure S8. Analysis of the Cx3cl1 project. PCR amplification of the genomic region of interest with (a) Cx3cl1-F1 and Cx3cl1-R1 primers (1483-bp amplicon) and (b) LoxPF and LoxPR primers (835-bp amplicon) from biopsies taken from the F0 animals. (c) The panels show the sequencing of PCR amplicon obtained from animal Cx3cl1-10 with Cx3cl1-F1 and Cx3cl1-R1. LoxP sequences are highlighted in blue. (d) The table details the F0 animals obtained. The ID and outcome of PCR analysis of the region of interest, as well as the conclusion for each individual are shown. Three founders are mated for cKO allele transmission (LoxP PCR positive and sequence of complex mosaic). PCR amplification of region of interest with (e) Cx3cl1-F1 and Cx3cl1-R1 primers (1483-bp amplicon) and LoxPF and LoxPR primers (835-bp amplicon) from biopsies taken from Cx3cl1-10’s offspring. (f) The table details the first litter obtained by mating Cx3cl1-10 with a WT mouse. The ID, outcome of sequencing the region of interest, copy counting of the region of interest and the conclusion for each individual are shown. (g) The panel shows an alignment of the sequencing data obtained from Cx3cl1-10.1a. Blue 5′homology arm; orange universal sequences for diagnostics; green critical region with exon in capitals; red loxP sites; grey 3′homology arm. (a, b, e) Animal IDs are shown. + is positive control amplified from an unrelated (a) WT, (b) conditional floxed animal. L1 = 1 kb DNA molecular weight ladder (thick band is 3 kb). Sequencing data showing examples of illegitimately repaired conditional alleles are shown in Additional file 3: Figure S2o and p. (PNG 1075 kb) [file 12915_2018_530_MOESM9_ESM.png]

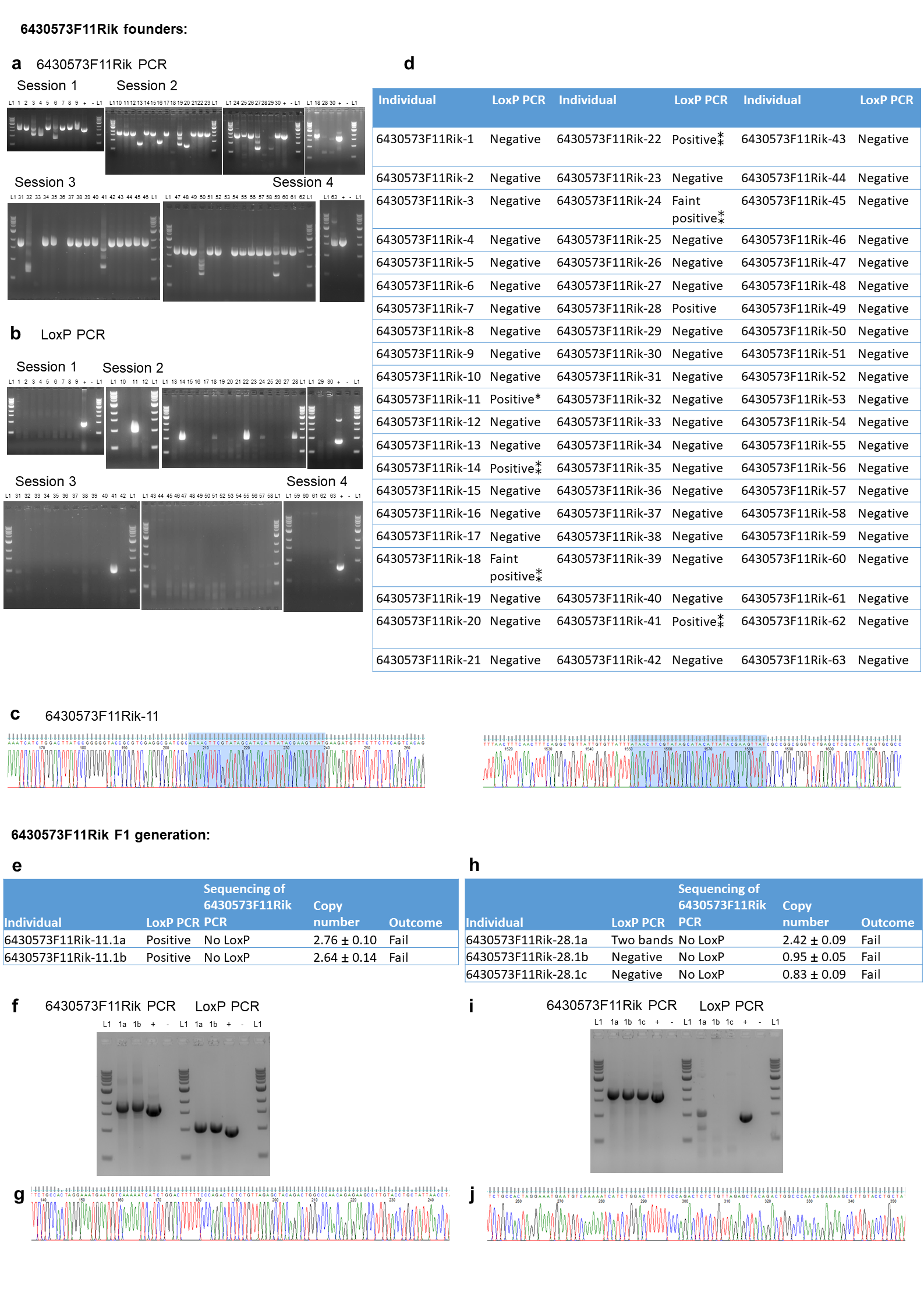

Supplement: Supplementary file 10 — Figure S9. Analysis of the 6430573F11Rik project. PCR amplification of genomic DNA of (a) F0 animals, (f) 6430573F11Rik-11’s offspring or (i) 6430573F11Rik-28’s offspring with (a, f) 6430573F11Rik-F3 and 6430573F11Rik-R2 (1721-bp amplicon) and (b, f) LoxPF and LoxPR (999-bp amplicon). Sequencing of PCR amplicons from (c) 6430573F11Rik-11 and (g) 6430573F11Rik-11.1a with 6430573F11Rik-F3 and 6430573F11Rik-R2. LoxPs are in blue. ID, outcome of PCR analysis and conclusion for (d) each F0 animal and (e) the first litter obtained by mating 6430573F11Rik-11 with a WT mouse. Two founders were mated for cKO GLT. *Mated; ⁑no evidence of loxP in 6430573F11Rik amplicon, suggesting donor integrated randomly (6430573F11Rik-28 sequence trace in Additional file 3: Figure S2q). (g) Only WT sequence is found, indicating random donor insertion. (f, i) Animal IDs are shown. + is positive control from unrelated WT and conditional floxed animal for 6430573F11Rik and LoxP PCR, respectively. L1 = 1 kb DNA molecular weight ladder (thick band is 3 kb). (h) First litter obtained by mating 6430573F11Rik-28 with a WT mouse. ID, outcome of sequencing and copy counting of the region of interest and the conclusion for each individual. (j) Sequencing of amplicons obtained with 6430573F11Rik-F3 and 6430573F11Rik-R2 and 6430573F11Rik-28.1a. Only WT sequence is found, indicating random donor insertion. Sequencing of deletion allele in founder 6430573F11Rik-6, summary of analysis of F1 animals derived from 6430573F11Rik-6 and transmitted deletion allele are shown in Additional file 3: Figure S2r, s and t. (PNG 1011 kb) [file 12915_2018_530_MOESM10_ESM.png]

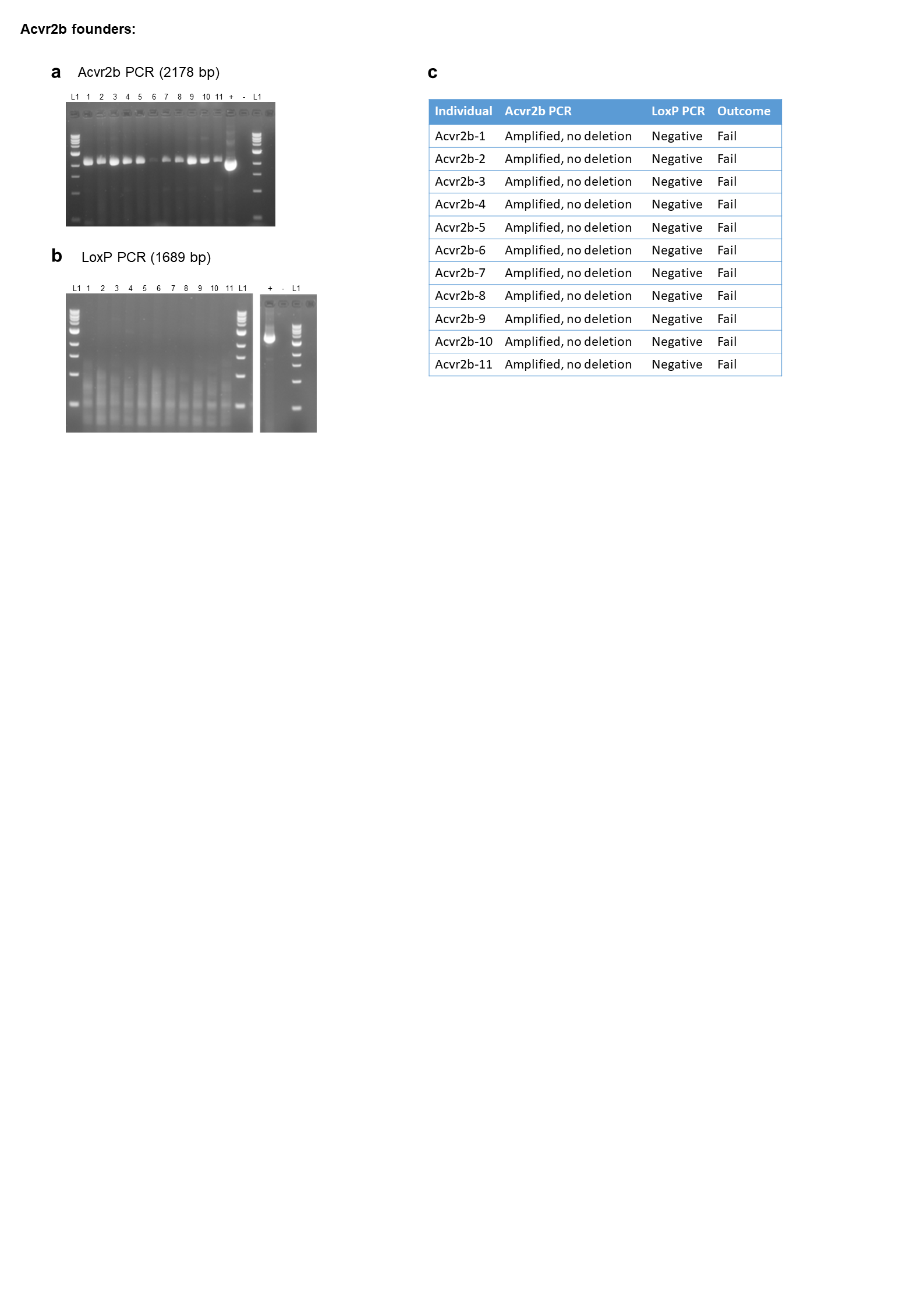

Supplement: Supplementary file 11 — Figure S10. Analysis of the Acvr2b project. The figure shows the PCR amplification of the genomic region of interest with (a) Acvr2b-F1 and Acvr2b-R1 primers (2178 bp) and (b) LoxPF and LoxPR primers (1689 bp) from biopsies taken from the F0 animals. (a, b) Animal IDs are shown. + is positive control amplified from an unrelated (a) WT, (b) conditional floxed animal. L1 = 1 kb DNA molecular weight ladder (thick band is 3 kb). (c) The table details the F0 animals obtained. The ID and outcome of PCR analysis of the region of interest as well as the conclusion for each individual are shown. (PNG 255 kb) [file 12915_2018_530_MOESM11_ESM.png]

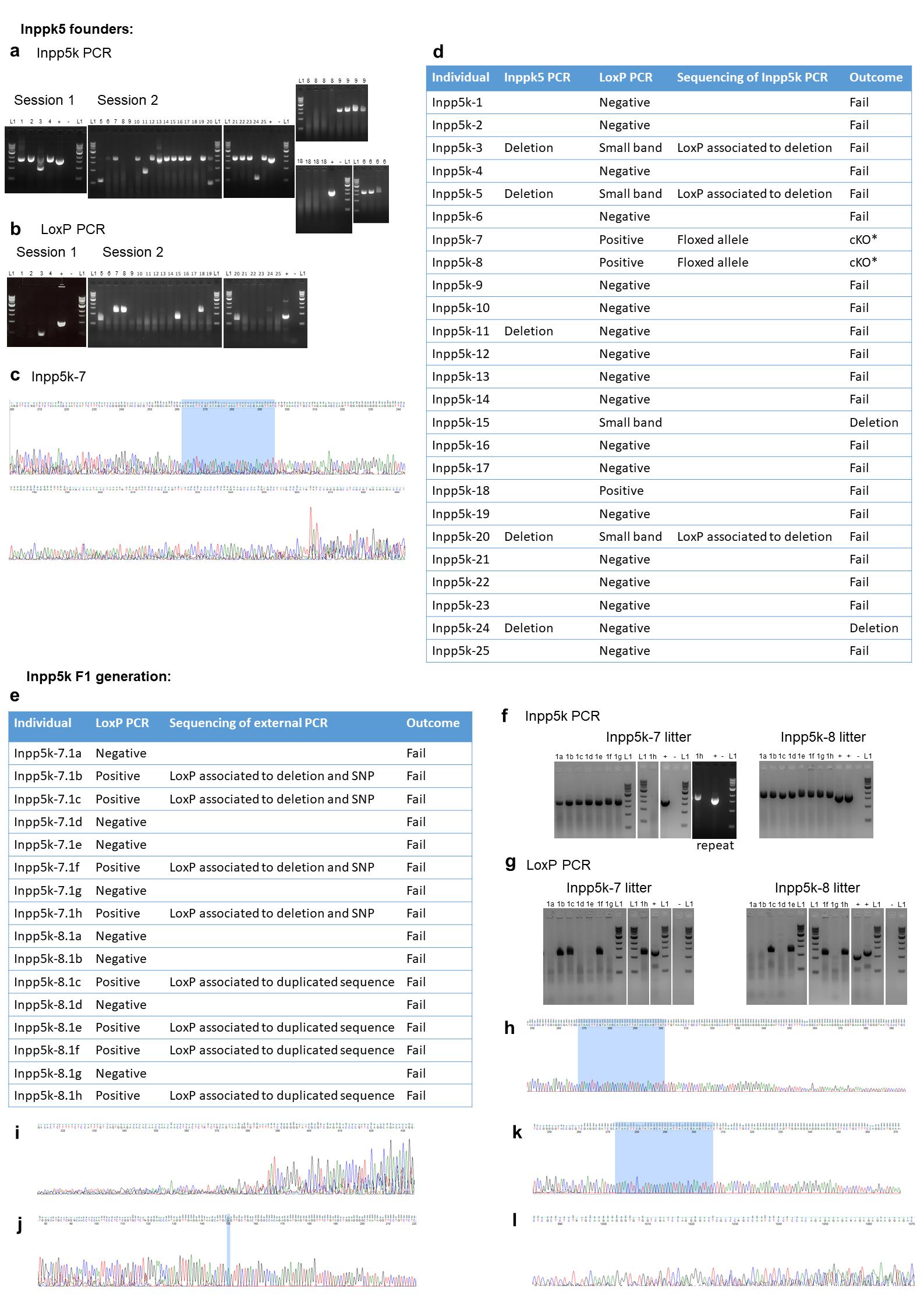

Supplement: Supplementary file 12 — Figure S11. Analysis of the Inpp5k project. The figure shows the PCR amplification of the genomic region of interest with (a) Inpp5k-F1 and Inpp5k-R1 primers (1705-bp amplicon) and (b) LoxPF and LoxPR primers (1194-bp amplicon) from biopsies taken from the F0 animals. Animal IDs are shown. + is positive control amplified from an unrelated WT and conditional floxed animal for the Inpp5k and LoxP PCR, respectively. L1 = 1 kb DNA molecular weight ladder (thick band is 3 kb). (c) Sequencing chromatogram of PCR amplicons obtained from Inpp5k-7 with Inpp5k-F1 and Inpp5k-R1. LoxP sequence is highlighted in blue. (d) The table details the F0 animals obtained. The ID, outcome of PCR analysis of the region of interest and the conclusion for each individual are shown. Two founders are mated for cKO allele transmission (LoxP PCR positive and sequence of complex mosaic). *Mated as loxP presence confirmed by sequencing of Inpp5k PCR amplicon. (e) First litter obtained by mating Inpp5k-7 and Inpp5k-8 with a WT mouse. The ID, outcome of sequencing the region of interest and the conclusion for each individual are shown. PCR amplification of region of interest with (f) Inpp5k-F1 and Inpp5k-R1 primers (1705-bp amplicon) and (g) LoxPF and LoxPR primers (1194-bp amplicon) from biopsies taken from Inpp5k-7’s and Inpp5k-8’s offspring. Animal IDs are shown. + is positive control amplified from an unrelated WT and conditional floxed animal for the Inpp5k and LoxP PCR, respectively. L1 = 1 kb DNA molecular weight ladder (thick band is 3 kb). Panels illustrate the sequencing data from amplicons obtained from Inpp5k-7.1b (h, i, j) and Inpp5k-8.1c (k, l) genomic DNA with (h, i, k) Inpp5k-F1 and (j, l) Inpp5k-R1 primers. (PNG 877 kb) [file 12915_2018_530_MOESM12_ESM.png]

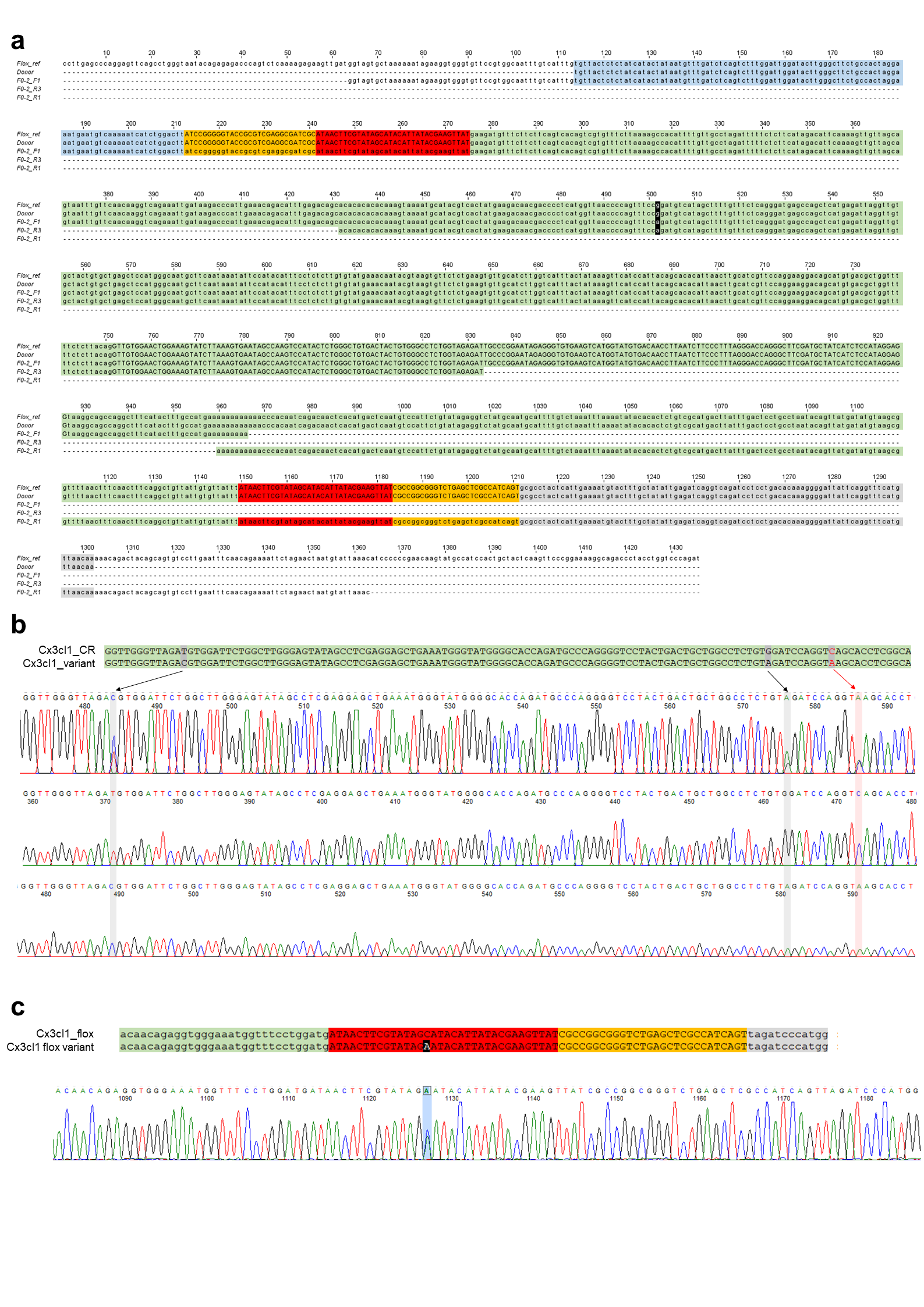

Supplement: Supplementary file 13 — Figure S12. Examples of unexpected point mutations in the F0 animals obtained from the co-injection of CRISPR/Cas9 reagents and lssDNA in 6430573F11Rik (a) and Cx3cl1 (b and c) projects. Blue 5′ homology arm; orange universal sequences for diagnostics; green critical region with exon in capitals; red loxP sites; grey 3′ homology arm. Unexpected point mutations are detected by Sanger sequencing of amplicons generated with primers external to the donor; (a) shows one intronic SNP in floxed critical region, (b) shows two intronic nucleotide changes (black arrows, grey highlight) and one coding nucleotide change (red arrow, pink highlight) which was found associated with (c) SNP in 3’ loxP site. Mutations are highlighted on the sequence alignment (a) and seen on the sequence chromatograms (b and c). (PNG 1332 kb) [file 12915_2018_530_MOESM13_ESM.png]

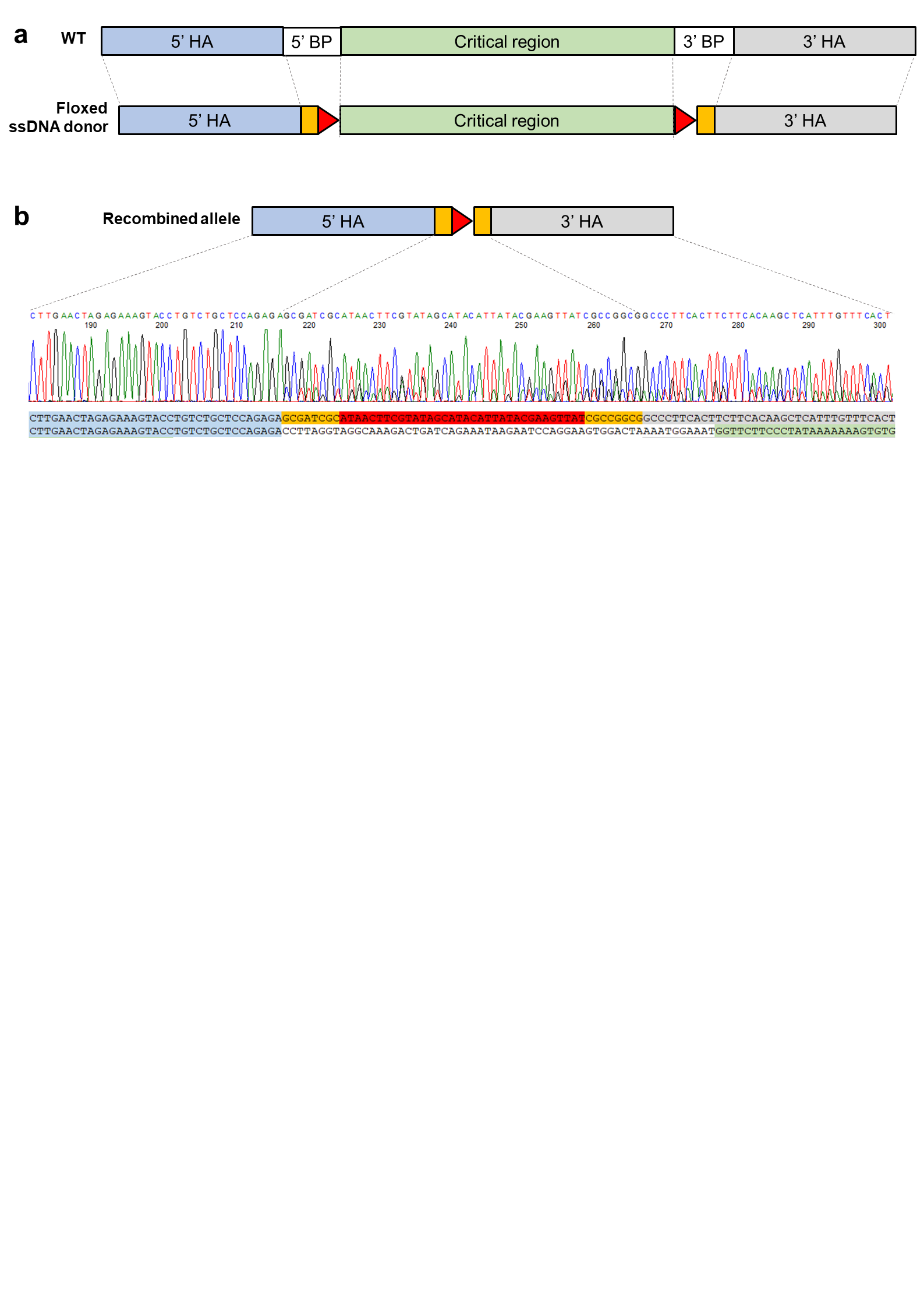

Supplement: Supplementary file 14 — Figure S13. Unexpected outcome of CRISPR/Cas9-aided mutagenesis. The figure illustrates an example of a rearranged allele obtained from the co-injection of CRISPR/Cas9 reagents and lssDNA to generate a conditional Ikzf2 allele. Panel (a) shows the design of the lssDNA donor compared to the WT sequence. HA homology arm, BP breakpoint (genomic sequence removed in the intended floxed allele). Panel (b) shows sequencing of an F1 (Ikzf2–2.1e) that bears a recombined allele where the critical region and a loxP site are lost (allele with major representation) and a WT allele (with minor representation). (PNG 309 kb) [file 12915_2018_530_MOESM14_ESM.png]

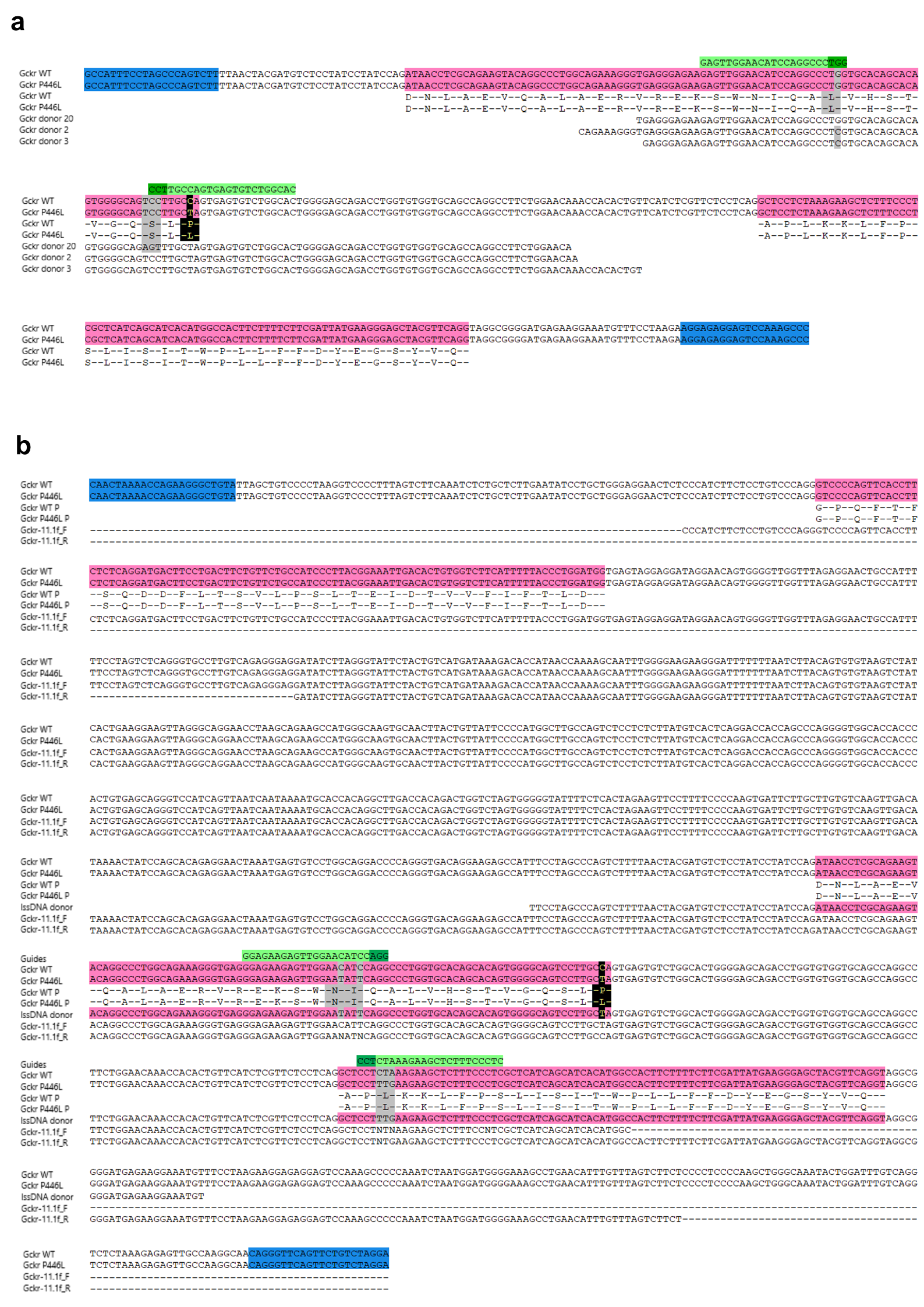

Supplement: Supplementary file 15 — Figure S14. Design of a GckrP446L point mutation. Figure illustrates the changes designed at the nucleotide and proteomic levels with the mutagenesis strategy employing (a) oligonucleotides and (b) lssDNA. Coding sequences are highlighted in pink, engineered P446L change is highlighted in black with yellow text, silent mutations are highlighted in grey and sgRNA sequences are highlighted in green. Primers external to the donors employed for mutant analysis are also shown in blue and detailed in Additional file 1: Table S1. (PNG 1857 kb) [file 12915_2018_530_MOESM15_ESM.png]

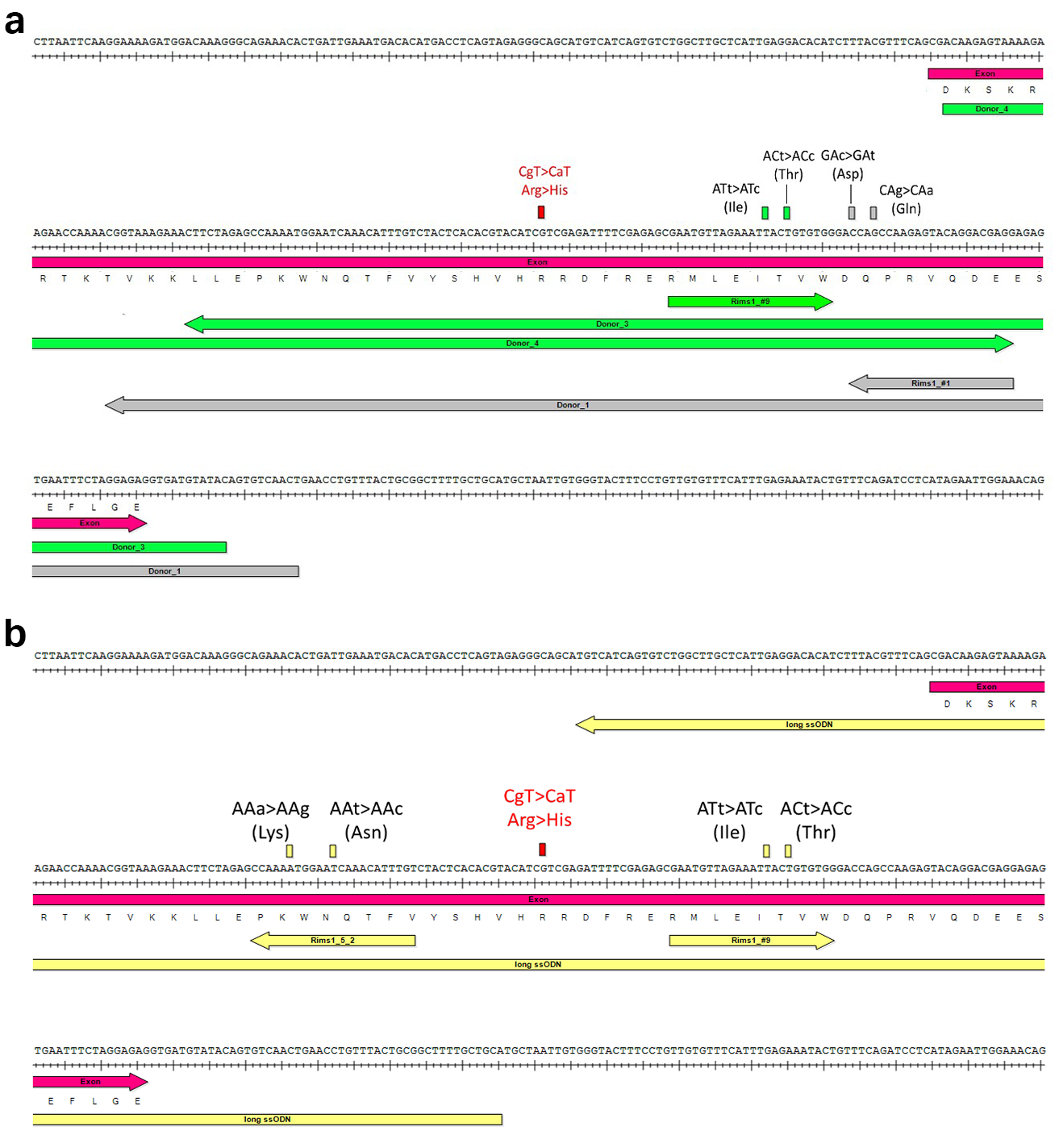

Supplement: Supplementary file 17 — Figure S15. Design of a Rims1R655H point mutation. The figure illustrates the changes designed at the nucleotide and proteomic levels with the mutagenesis strategy employing (a) oligonucleotides and (b) lssDNA. Coding sequences are translated into protein sequences above annotated exon. Note that the region containing Rims1 is not entirely accurate in the GRCm38 assembly. We have re-sequenced this region prior to designing of the mutant (primers shown in Additional file 1: Table S1). (PNG 521 kb) [file 12915_2018_530_MOESM17_ESM.png]

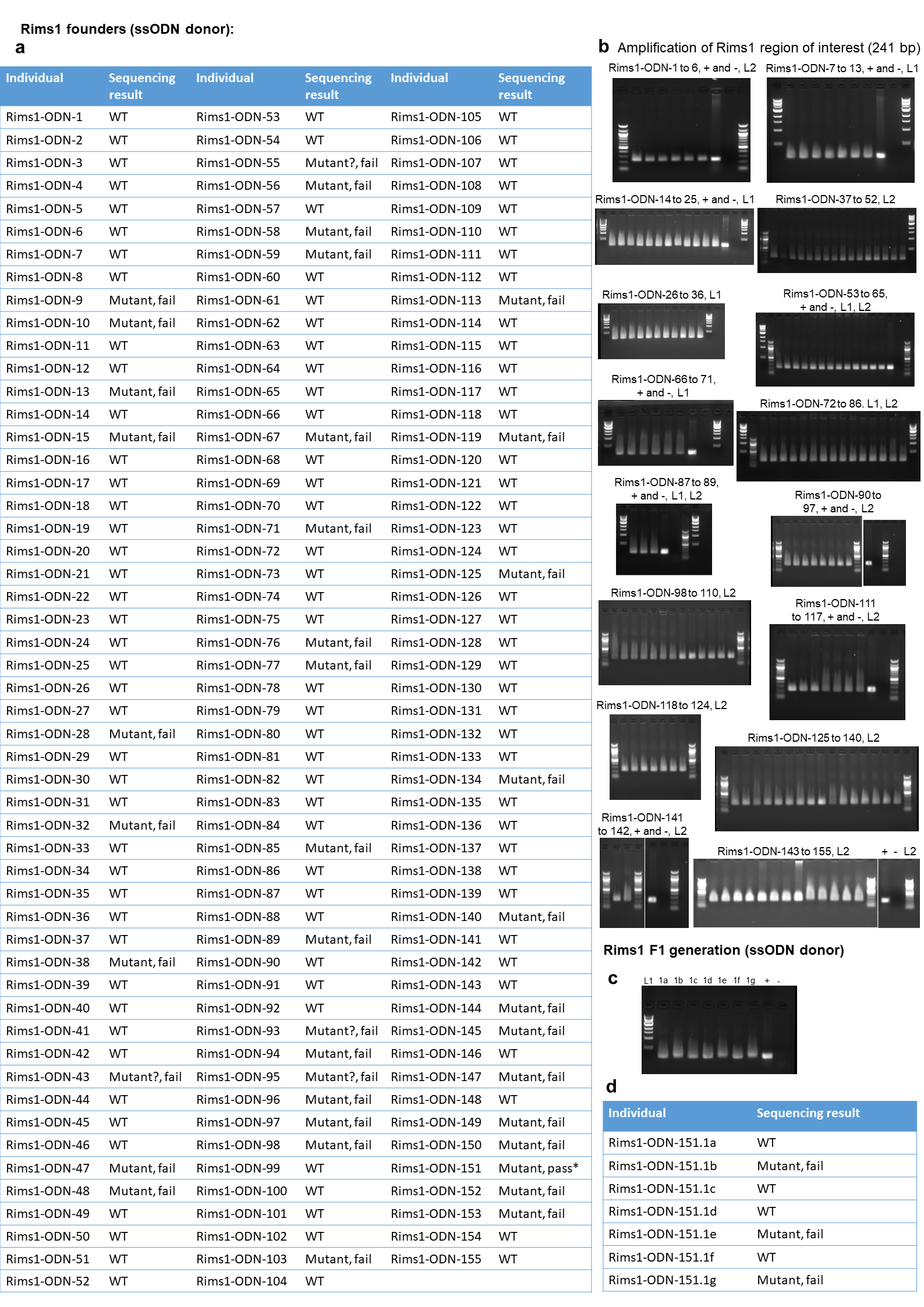

Supplement: Supplementary file 18 — Figure S16. Generation of a point mutation in Rims1 with ssODN donors. (a) The table details the F0 animals obtained for generation of Rims1 mutant with ssODN donors. The ID and outcome of sequencing the region of interest, as well as the conclusion for each individual are shown. (b) PCR amplification of region of interest with Rims1-F1 and Rims1-R1 primers (241 bp) from biopsies taken from the F0 animals. Sequences of Rims1-ODN-151 mosaic and of sub-cloned amplicons are shown in Additional file 3: Figure S2u and v, demonstrating the presence of the desired mutation in this animal that was therefore mated. (c) PCR amplification of region of interest with Rims1-F1 and Rims1-R1 primers (241 bp) from biopsies taken from Rims1-ODN-151’s offspring. Animal IDs are shown. + is positive control amplified from an unrelated WT animal. L1 = 1 kb DNA molecular weight (thick bands are 3 kb); L2 = 100 bp DNA molecular weight ladder (thick bands are 1000 and 500 bp). (d) The table details the first litter obtained by mating Rims1-ODN-151 with a WT mouse. The ID, outcome of sequencing the region of interest and copy counting of the region of interest as well as the conclusion for each individual are shown. Sequencing of Rims1-ODN-151.1g is shown in Additional file 3: Figure S2w and illustrates the failure of transmission of the desired allele. (PNG 893 kb) [file 12915_2018_530_MOESM18_ESM.png]

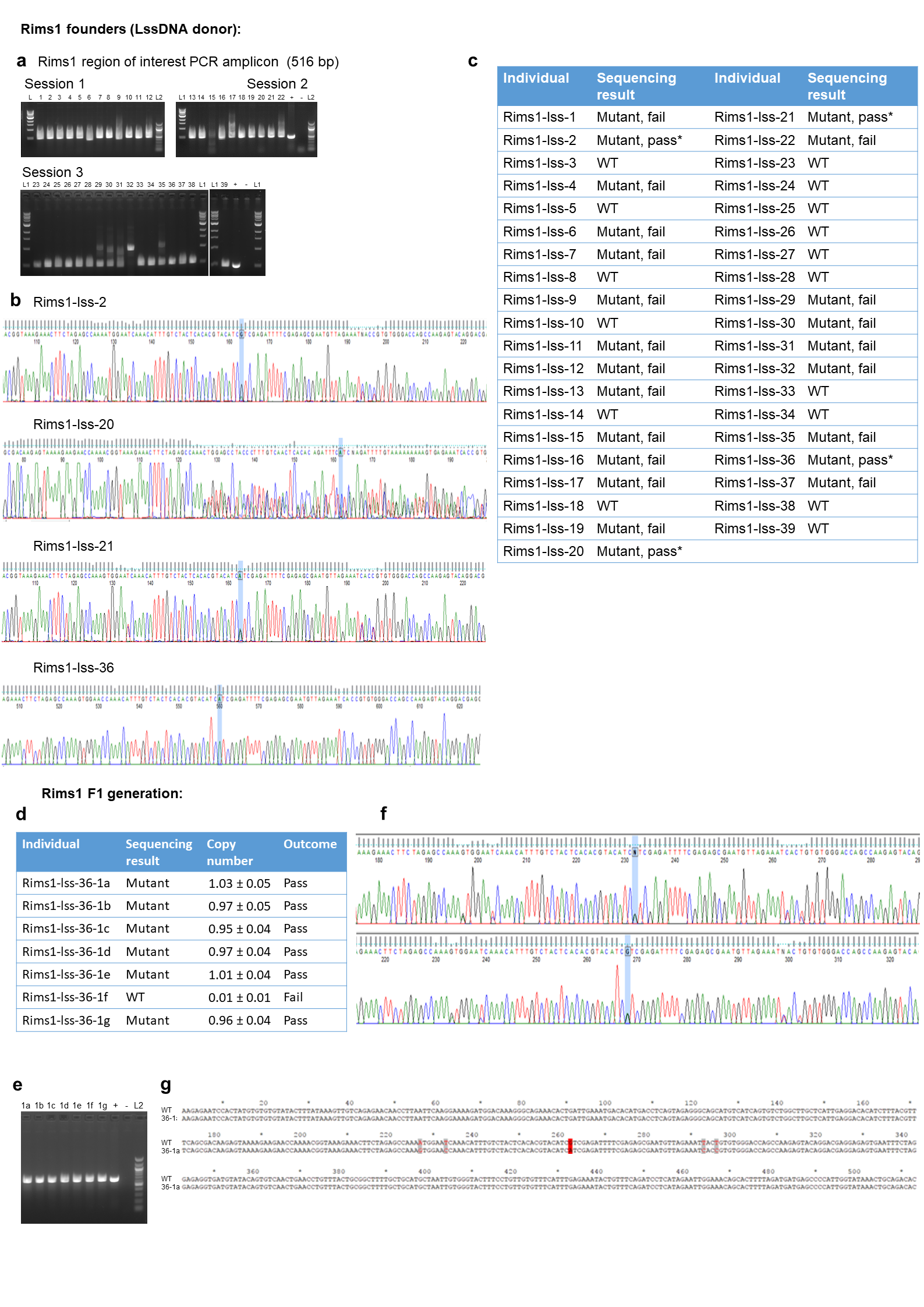

Supplement: Supplementary file 19 — Figure S17. Generation of a point mutation in Rims1 with a lssDNA donors. (a) PCR amplification of region of interest with Rims1-F2 and Rims1-R2 primers (647 bp) from biopsies taken from the F0 animals. Animal IDs are shown. + is positive control amplified from an unrelated WT animal. L1 = 1 kb DNA molecular weight ladder (thick band is 3 kb). (b) Sequencing of amplicon obtained from the Rims1-lss-2, Rims1-lss-20, Rims1-lss-21 and Rims1-lss-36 animals: point mutation is observed (blue highlight) when sequencing the Rims1-F2 primer. (c) The table details the F0 animals obtained for generation of Rims1 mutant with lssDNA donors. The ID, outcome of sequencing the region of interest and the conclusion for each individual are shown. (d) The table details the first litter obtained by mating Rims1-lss-36 with a WT mouse. The ID, outcome of sequencing the region of interest, copy counting of the region of interest and conclusion for each individual are shown. (e) PCR amplification of region of interest with Rims1-F3 and Rims1-R3 primers (647 bp) from biopsies taken from Rims-lss-36’s offspring. Animal IDs are shown. + is a positive control amplified from an unrelated WT animal. L2 = 100 bp DNA molecular weight ladder (thick bands are 1000 and 500 bp). (f) Sequencing of amplicon obtained from Rims1-lss-36.1a, legitimate repair observed (blue highlight) when sequencing both directions (Rims1-F3 and Rims1-R3 primers). (g) Alignment of Rims1-lss-36-1a offspring, legitimate repair aligned against WT allele. R655H coding change highlighted in red. Grey background with red text highlights silent mutations introduced by long donor. (PNG 1287 kb) [file 12915_2018_530_MOESM19_ESM.png]
