# Supplementary material for: Application of long single-stranded DNA donors in genome editing: generation and validation of mouse mutants
Source: BMC Biol. 2018 Jun 21;16:70. doi: 10.1186/s12915-018-0530-7 (PMC6011369; doi:10.1186/s12915-018-0530-7)

1. Alignment of sequencing data of Syt7 PCR amplicon from F1 Syt7-8.1f and Syt7-4.1d with amplified and sequenced with primers Syt7-F1 and Syt7-R1. To get full coverage of the allele, the PCR amplicons from Syt7-8.1f were sub-cloned and sequenced with both Syt7-F1 and Syt7-R1. In blue: 5’ homology arm; in orange: universal sequences for diagnostics; green: critical region with exon in capitals; red: loxp sites and grey: 3’ homology arm.


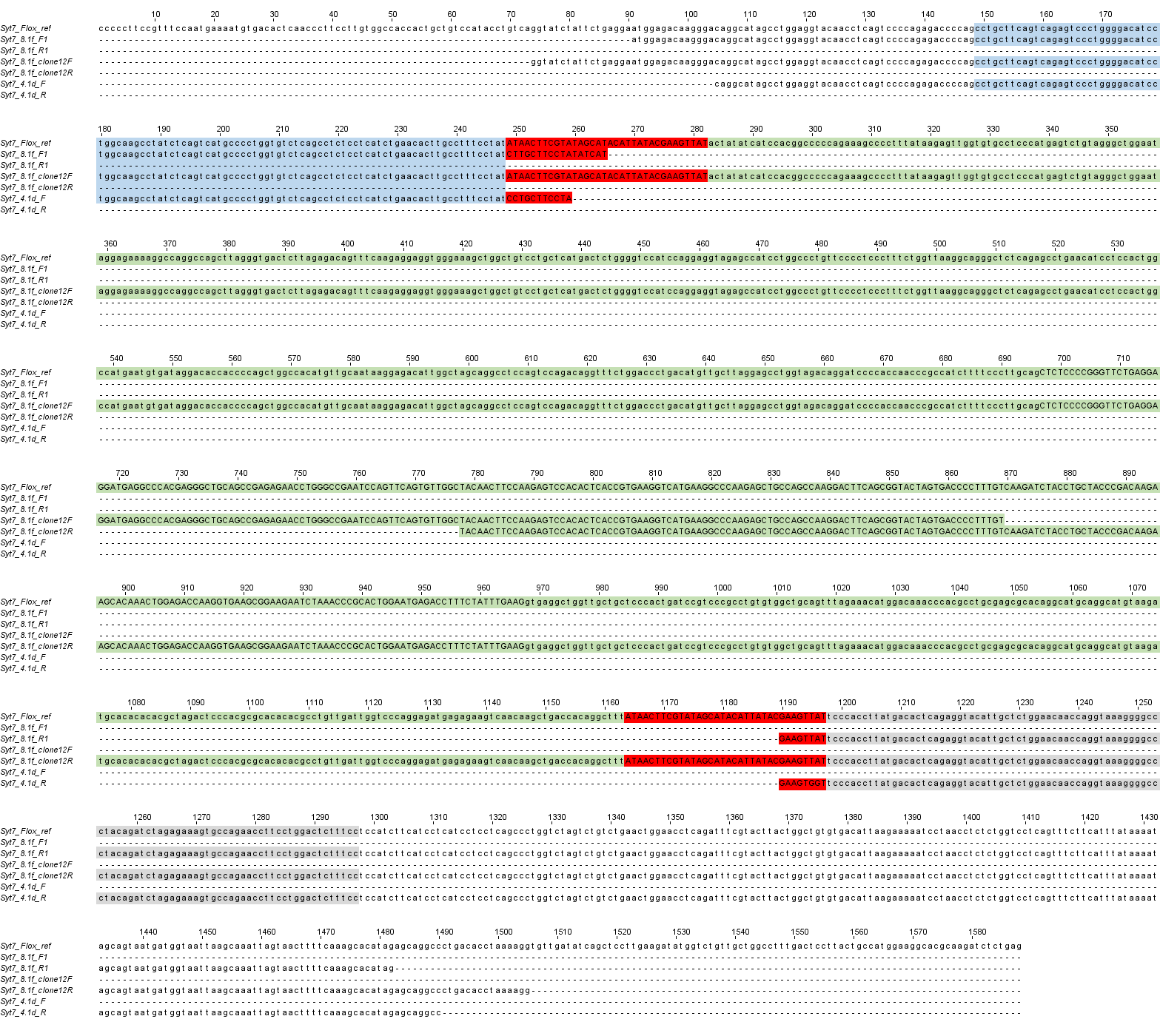


1. Sequencing of Ikzf2 PCR amplicon from Ikzf2-4 with Ikzf2-F3 primer showing deletion.


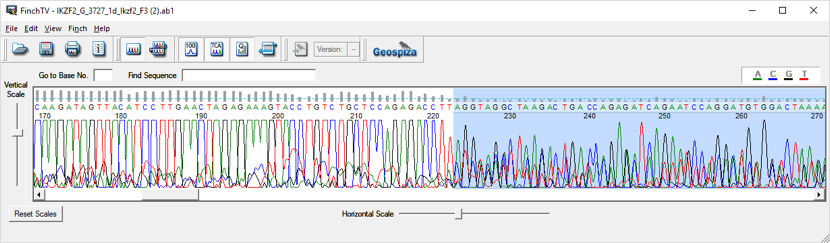


1. Sequencing of Ikzf2 PCR amplicon from Ikzf2-8 with Ikzf2-F3 primer showing deletion.


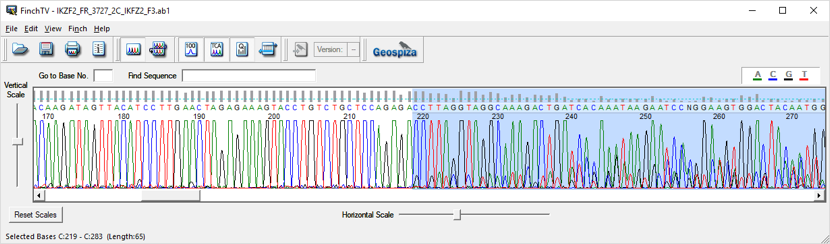


1. Alignment of sequencing data of Ikzf2 PCR amplicons from F_1_ Ikzf2-2.1a using external primers (Ikzf2-F3 and Ikzf2-R2) and generic loxP-specific primers (LoxPF and LoxPR). In blue: 5’ homology arm; in orange: universal sequences for diagnostics; green: critical region with exon in capitals; red: loxp sites and grey: 3’ homology arm.


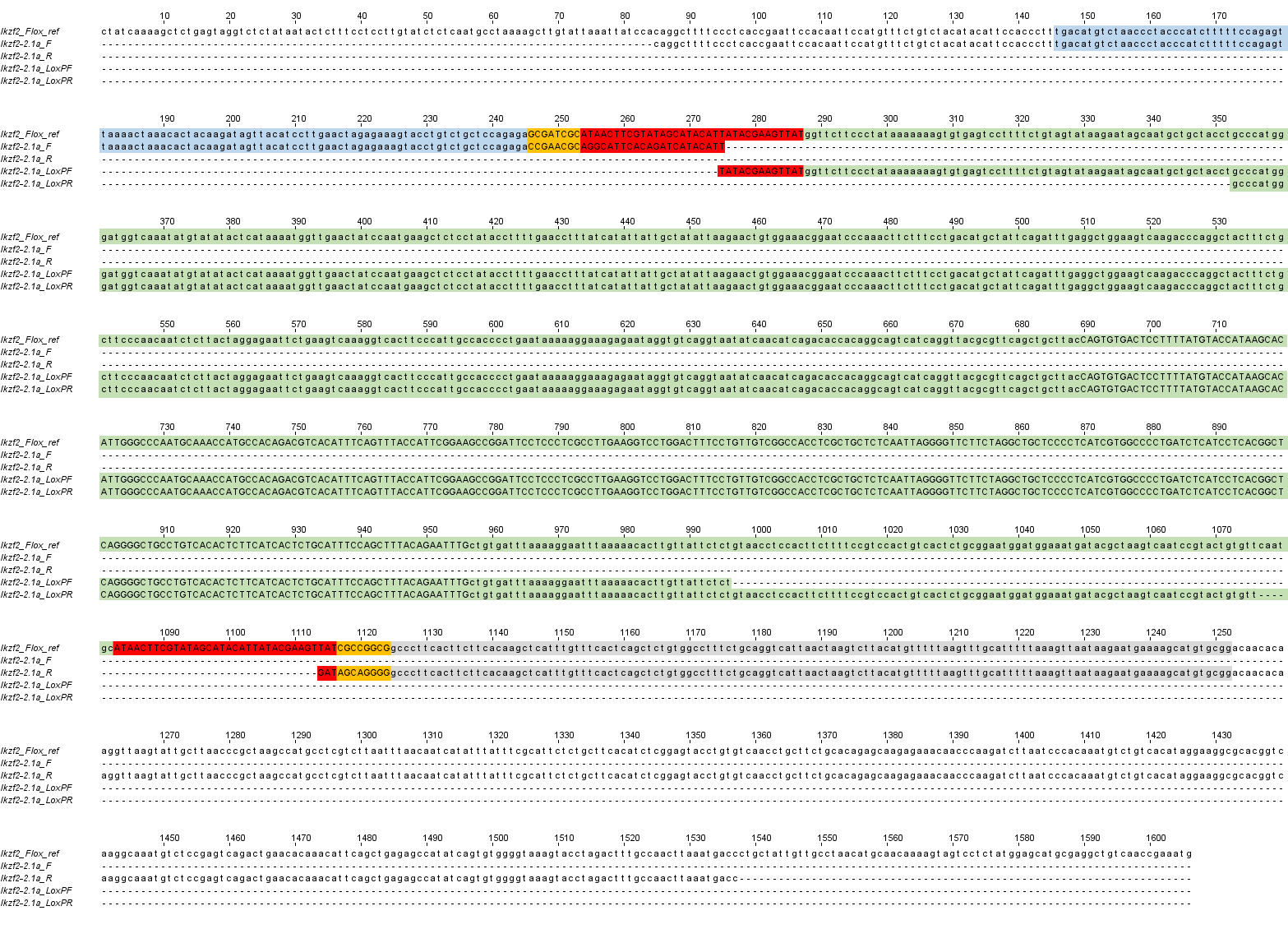


1. Sequencing of Syt4 PCR amplicon from Syt4-17 with Syt4-R2 primer showing deletion highlighted in blue.


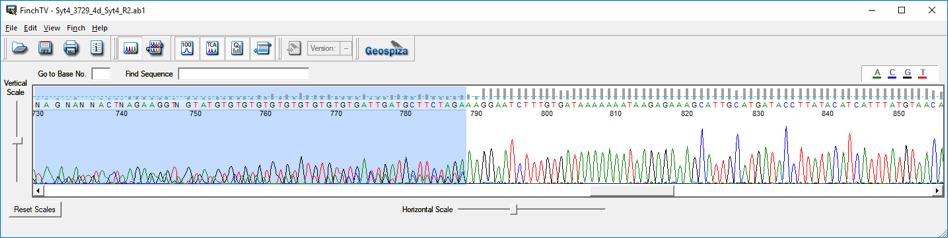


1. Analysis with Syt4 PCR of F1s from founder Syt4-17. L1 ladder (= 1 kb DNA molecular weight ladder (thick band is 3 kb)) is employed.


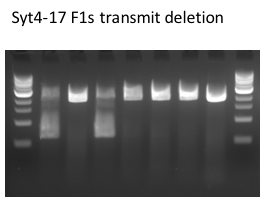


1. Sequencing of Syt4 PCR amplicon from Syt4-17.1a with Syt4-F4 primer showing deletion highlighted in blue.


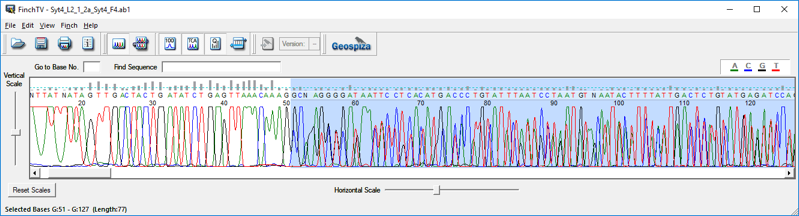


1. Sequencing of Syt4 PCR amplicon from founder Syt4-28 with Syt4-F4 primer showing deletion and possible insertion of loxp site highlighted in blue.


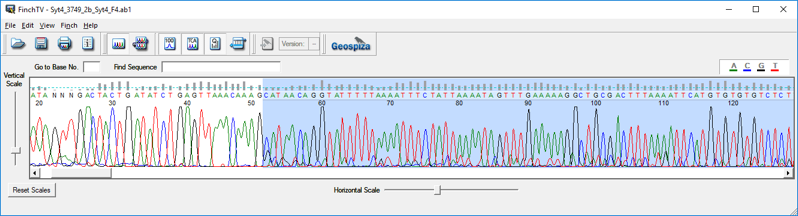


1. Sequencing of Syt4 PCR amplicon from an offspring of founder Syt4-28 with Syt4-F4 primer showing an illegitimate repair transmitted (loxP site is highlighted).


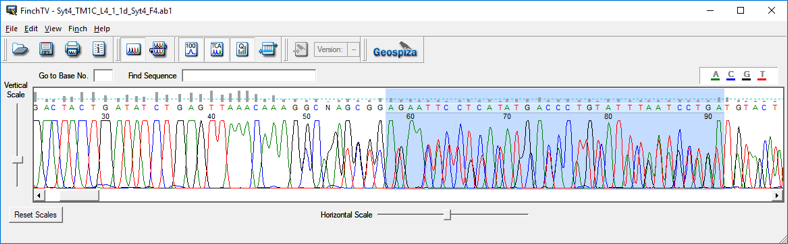


1. Sequencing of Syt4 PCR amplicon from founder Syt4-37 with Syt4-F4 and Syt4-R2 primers showing no evidence of loxP site integration.


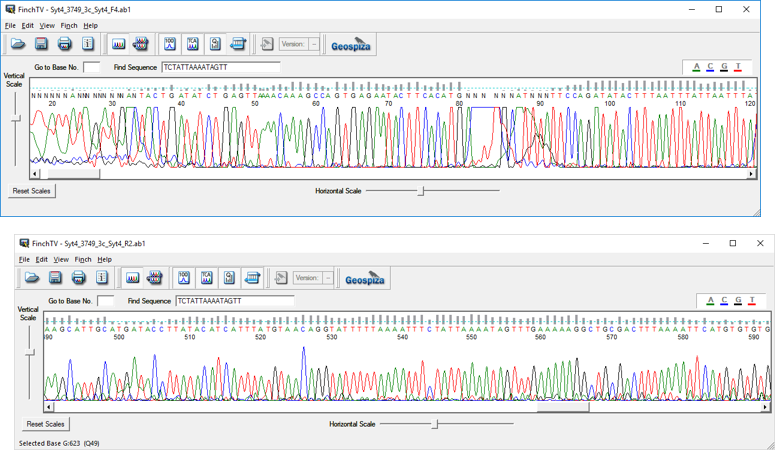


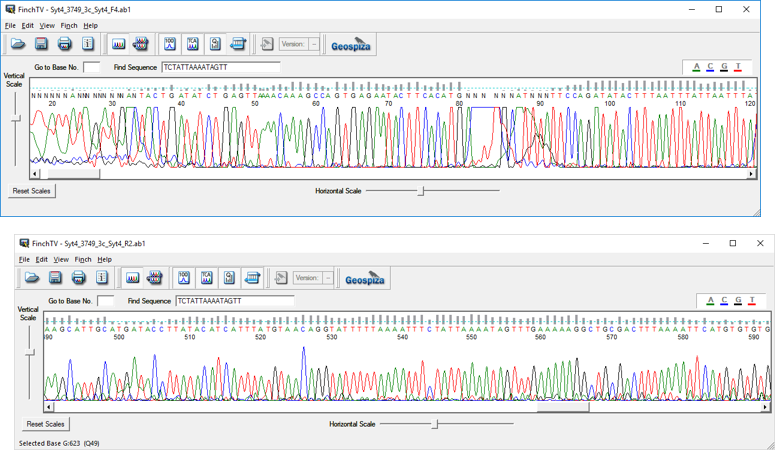


1. Alignment of sequencing data of Syt4 PCR amplicon from F1 Syt4-29.1a with primers Syt4-F2 and Syt4-R2 sequenced with Syt4-F4 (to bypass poly-repeated region) and Syt4-R1. To get full coverage of the allele, the PCR amplicons were sub-cloned and sequenced with both Syt4-R1 and Syt4-R3. In blue: 5’ homology arm; in orange: universal sequences for diagnostics; green: critical region with exon in capitals; red: loxp sites and grey: 3’ homology arm.


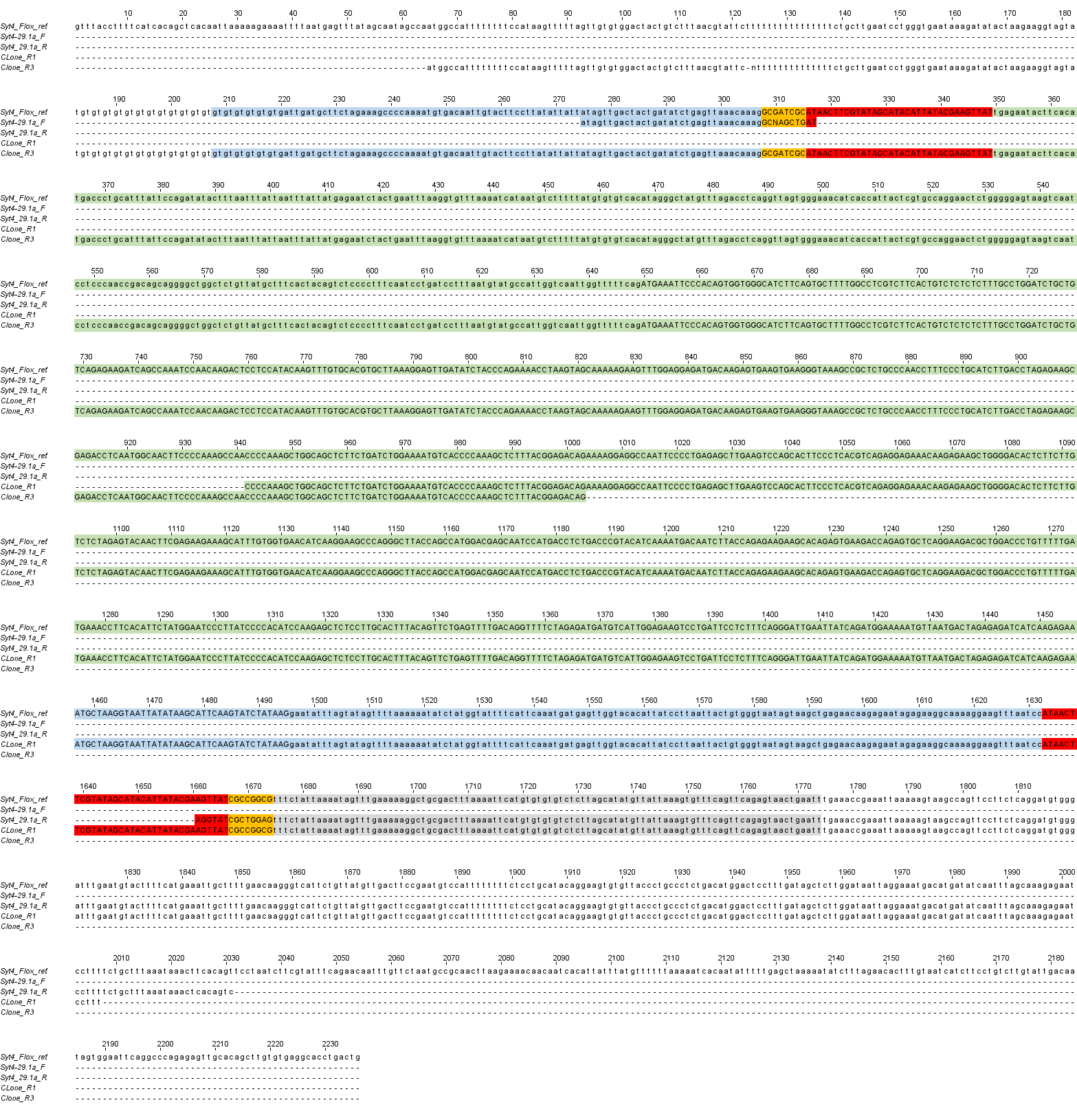


1. Sequencing of Usp45 PCR amplicon from Usp45-18.1a with Usp45-F1 primer showing a 5’ NHEJ event associated with the 3’ loxP site.


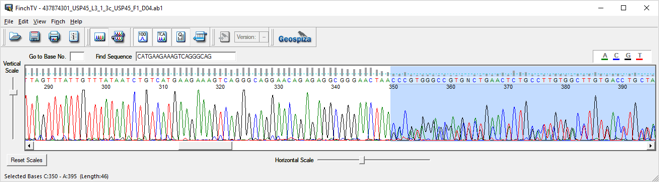


1. Sequencing of Usp45 PCR amplicon from Usp45-18.1b with Usp45-F1 primer showing a NHEJ event.


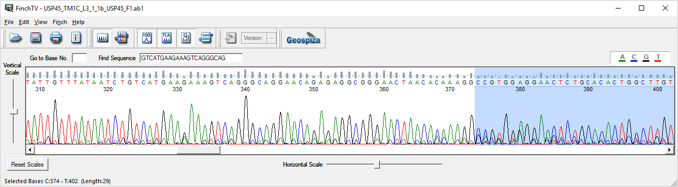


1. Sequencing of Rapgef5 PCR amplicon from founder Rapgef5-3 with Rapgef-F1 primer illustrating the break point of a deletion.


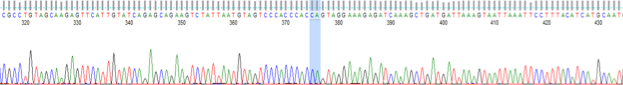


1. Sequencing of Cx3cl1 PCR amplicon from founder Cx3cl1-14 with Cx3cl1-F2 and Cx3cl1-R3 primers showing loxP sites (highlighted in blue) and a single nucleotide change (blue arrow).


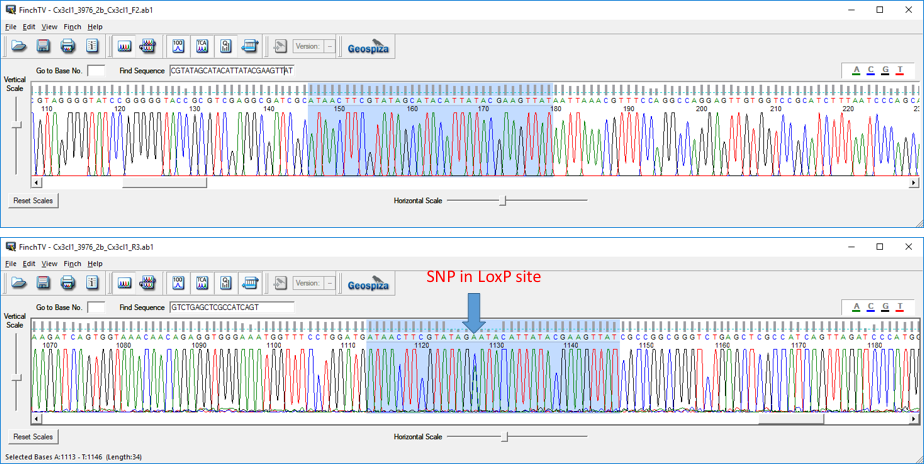


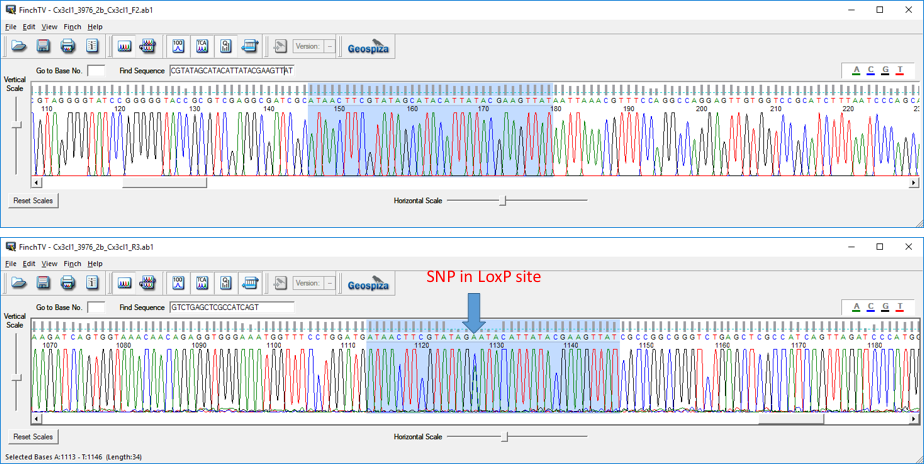


1. Sequencing of Cx3cl1 PCR amplicon from founder Cx3cl1-17 with LoxP-F and LoxP-R primers showing a deletion in the floxed allele.


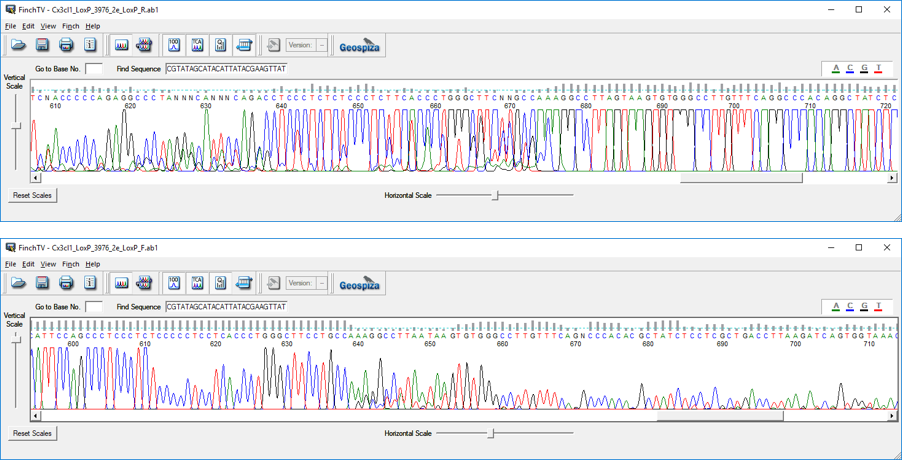


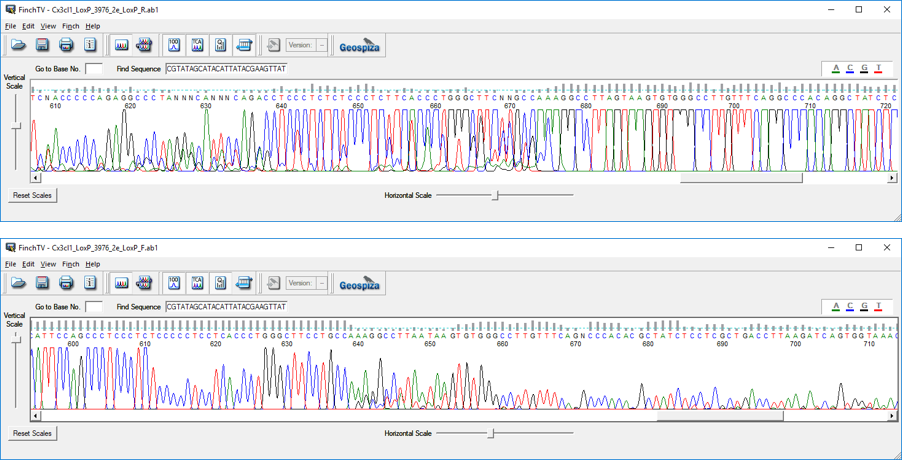


1. Sequencing of 6430573F11Rik PCR amplicon from founder 6430573F11Rik-28 with LoxP-F and LoxP-R primers -F and LoxP-R primers LoxP-F and LoxP-R primers showing a deletion in the floxed allele.


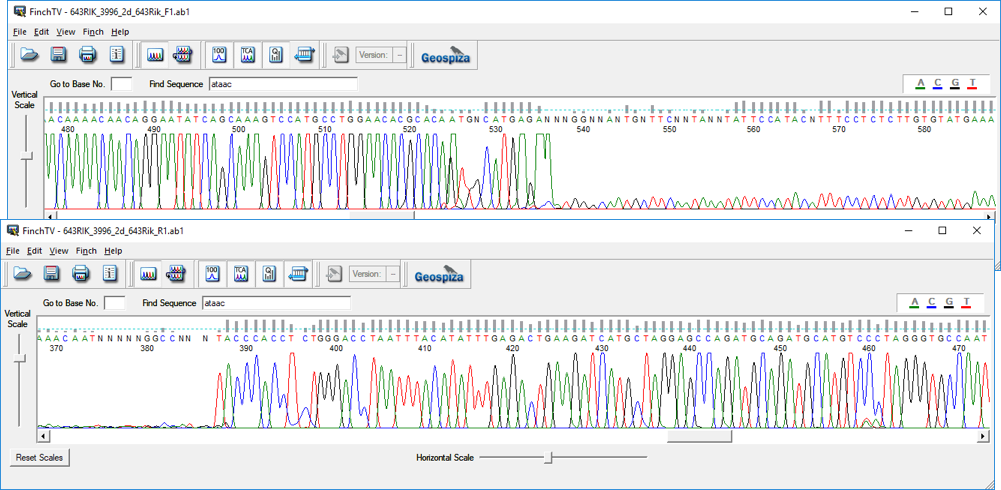


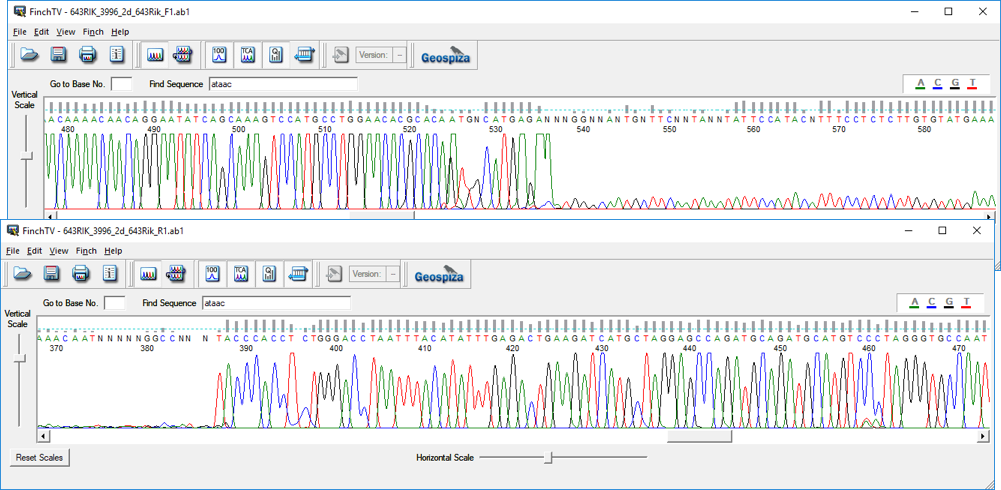


1. Sequencing of 6430573F11Rik PCR amplicon from founder 6430573F11Rik-6 with 6430573F11Rik-F and 6430573F11Rik-R primers showing a deletion.


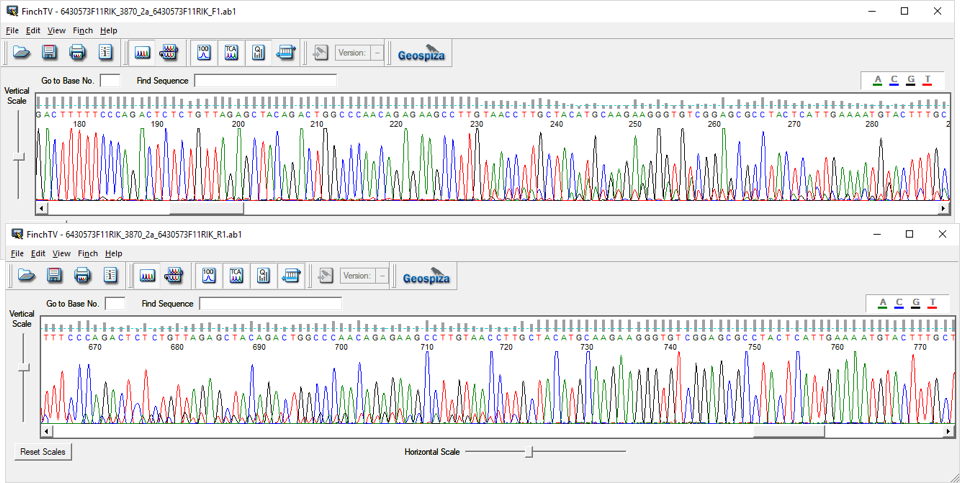


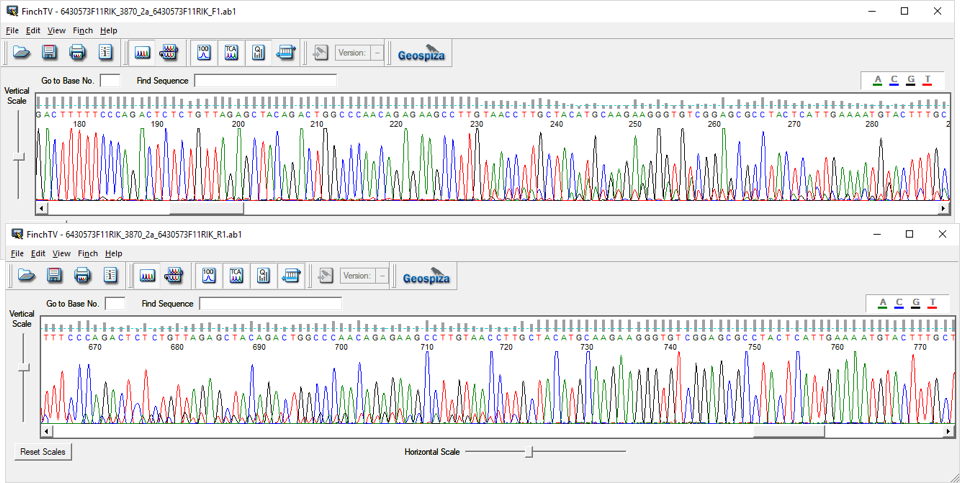


1. Breeding of founder 6430573F11Rik-6.

| Individual | LoxP PCR | Sequencing of external PCR | Copy number | Outcome |
| --- | --- | --- | --- | --- |
| 6430573F11Rik-6.1a | Negative | No deletion | 2.11 ± 0.09 | Fail |
| 6430573F11Rik-6.1b | Negative | Deletion | 1.05 ± 0.04 | Deletion |
| 6430573F11Rik-6.1c | Negative | Deletion | 1.01 ± 0.04 | Deletion |
| 6430573F11Rik-6.1d | Negative | Deletion | 1.06 ± 0.05 | Deletion |
| 6430573F11Rik-6.1e | Negative | No deletion | 1.97 ± 0.05 | Fail |
| 6430573F11Rik-6.1f | Negative | Deletion | 1.07 ± 0.04 | Deletion |
| 6430573F11Rik-6.1g | Negative | No deletion | 2.06 ± 0.07 | Fail |

1. Sequencing of 6430573F11Rik PCR amplicon from 6430573F11Rik-6.1b with 6430573F11Rik-F primer showing 925 nt deleted replaced by 5 nt insertion (highlighted).


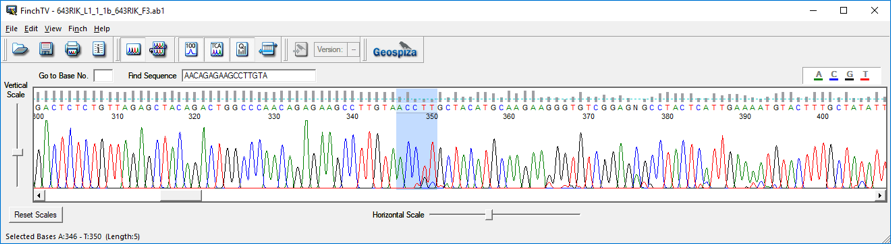


1. Sequencing of Rims1 PCR amplicon from founder Rims1-151 with Rims1-F1 primer showing mosaicism.


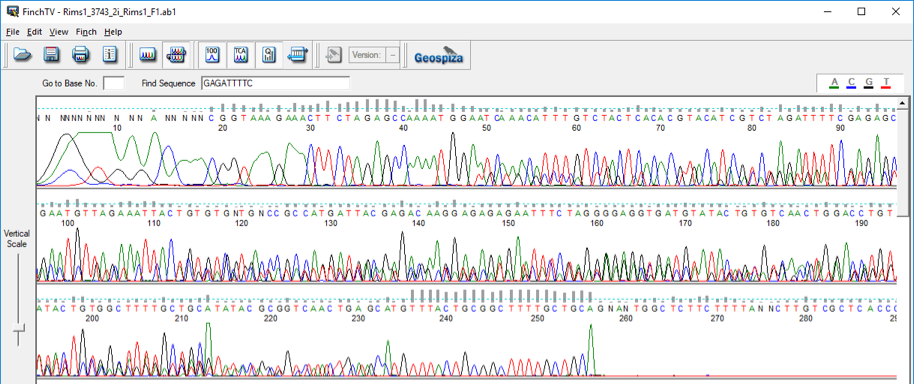


1. Sequencing of sub-cloned Rims1 PCR amplicon from founder Rims1-151 with Rims1-F1 primer showing mosaicism. Point mutation is highlighted.


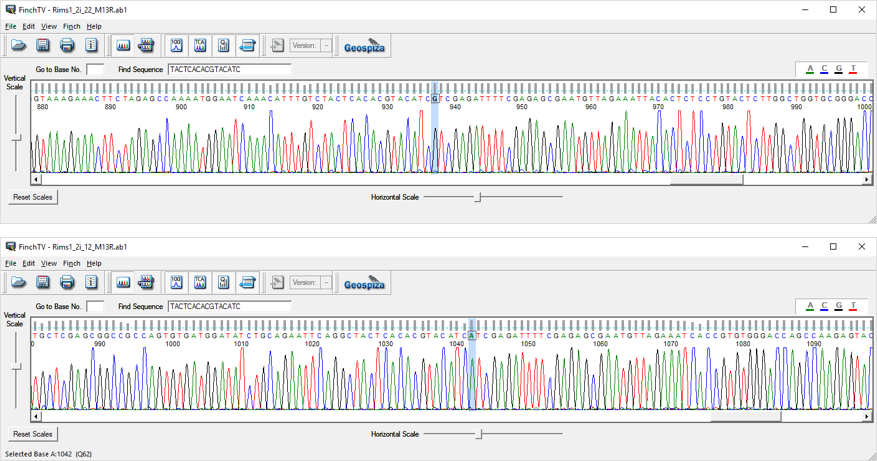


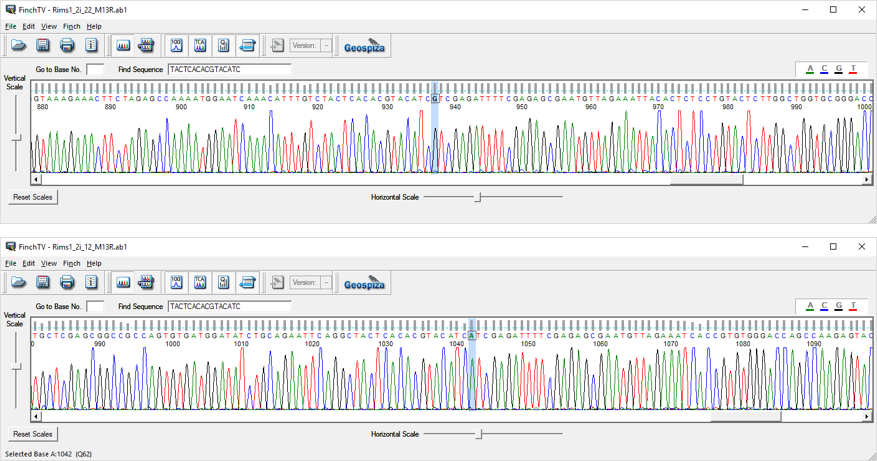


1. Sequencing of Rims1 PCR amplicon from Rims1-151.1g (Rims1-151 progeny) with Rims1-F1 primer showing R655H repair (the desired point mutation (highlighted), plus two silent mutations and a 44 nt insertion. This allele was also seen in subsequent litters.


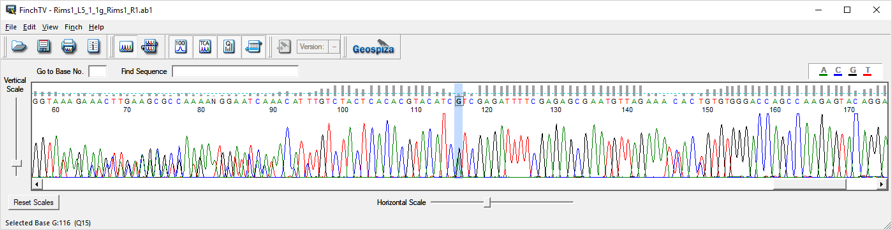

Supplement: Supplementary file 3 — Figure S2. Additional animal analysis information. (DOCX 19408 kb) [file 12915_2018_530_MOESM3_ESM.docx]
